# Hippocampal CA1 replay becomes less prominent but more rigid without inputs from medial entorhinal cortex

**DOI:** 10.1038/s41467-019-09280-0

**Published:** 2019-03-22

**Authors:** Alireza Chenani, Marta Sabariego, Magdalene I. Schlesiger, Jill K. Leutgeb, Stefan Leutgeb, Christian Leibold

**Affiliations:** 1 Department Biology II, Ludwig-Maximilians-Universität München, Martinsried, 82152 Germany; 2 Neurobiology Section and Center for Neural Circuits and Behavior, University of California, San Diego, La Jolla, 92093 CA USA; 3 Kavli Institute for Brain and Mind, University of California, San Diego, La Jolla, 92093 CA USA; 4 Bernstein Center for Computational Neuroscience Munich, Martinsried, 82152 Germany; 5 Present Address: Max-Planck Institute for Psychiatry, 80804 Munich, Germany; 6 Present Address: Department of Clinical Neurobiology, Medical Faculty of Heidelberg University and German Cancer Research Center (DKFZ), 69120 Heidelberg, Germany

## Abstract

The hippocampus is an essential brain area for learning and memory. However, the network mechanisms underlying memory storage, consolidation and retrieval remain incompletely understood. Place cell sequences during theta oscillations are thought to be replayed during non-theta states to support consolidation and route planning. In animals with medial entorhinal cortex (MEC) lesions, the temporal organization of theta-related hippocampal activity is disrupted, which allows us to test whether replay is also compromised. Two different analyses—comparison of co-activation patterns between running and rest epochs and analysis of the recurrence of place cell sequences—reveal that the enhancement of replay by behavior is reduced in MEC-lesioned versus control rats. In contrast, the degree of intrinsic network structure prior and subsequent to behavior remains unaffected by MEC lesions. The MEC-dependent temporal coordination during theta states therefore appears to facilitate behavior-related plasticity, but does not disrupt pre-existing functional connectivity.

## Abstract (web summary)

Medial entorhinal cortex (MEC) is involved in memory processes that entail the replay of sequential firing of hippocampal place cells during rest periods and during behaviour. Here, the authors show that MEC lesioned animals show intact replay after an epoch of rats running on a linear track, while replay during the behavioral epoch is reduced.

## Introduction

Population bursts of hippocampal neurons occur during sleep and immobility, often in association with hippocampal sharp waves^1,2^. Because immobility-related population bursts replay sequences of place cells that correspond to previous running trajectories in an environment^3^, replay was classically thought to reflect experiences that an animal had during previous episodes of running on a maze^3,4^, consistent with the hypothesis that replay supports memory consolidation processes^5–9^. However, place cell sequences can also be replayed in backward or mixed directions^4,10,11^, and there are reports that population bursts can express preplay of trajectories that the animal is about to follow in the future in a known environment^4,12^ and, although controversial^13^, even in a yet unknown environment^14,15^. These observations make the interpretation that population bursts are a substrate for memory consolidation less straightforward, but bursts might nonetheless be memory related. For example, correlations between sequences and future behavior could reflect prewired task-specific schemas that facilitate the formation of novel memory traces^16^.

In order to examine whether sequences during population bursts are experience-related, they are typically compared to neuronal activity patterns during behavior and, in particular, the patterns that occur during periods of running. During running, theta oscillations are the predominant brain state^1,17^ and spike-timing is organized such that the sequences of place fields that are traversed in an environment emerge in parallel in the same sequential order as time-compressed theta sequences within individual theta cycles^18,19^. According to one controversially discussed^20,21^ class of models^22,23^ theta sequences and sequence replay during sharp waves both result from the same pre-existing recurrent hippocampal connectivity. A second class of models^24,25^, in contrast, predicts that replay is a result of synaptic plasticity that is induced by intact theta sequences via spike-timing dependent plasticity^26–28^. One way to distinguish between the two model classes is to study the expression of sequence replay in animals with disrupted theta sequences. It has previously been show that spike-timing during theta states is strongly disrupted in rats with bilateral medial entorhinal cortex (MEC) lesions^29^. The MEC directly projects to the hippocampus and is one major source of information supporting spatial memory^30,31^. Nevertheless, place fields are retained in animals in which the MEC is either permanently lesioned or acutely inactivated using optogenetic or chemogenetic techniques^32,33^, even though they are less abundant, less stable and less precise than in controls^34^. The models explaining replay as a result of spike-timing dependent plasticity predict that sequences do not emerge during behavior in MEC-lesioned rats. In contrast, other scenarios in which replay is a consequence of any pre-existing connectivity predict that schema-related replay components, already encoded in the synaptic connections before the lesion, should remain unaffected. The analysis of replay and pattern activation in animals with MEC-lesions thus makes it feasible to probe both of the current views on hippocampal replay.

## Results

### MEC-lesions reduce mean co-activation strength

**Fig. 1:**
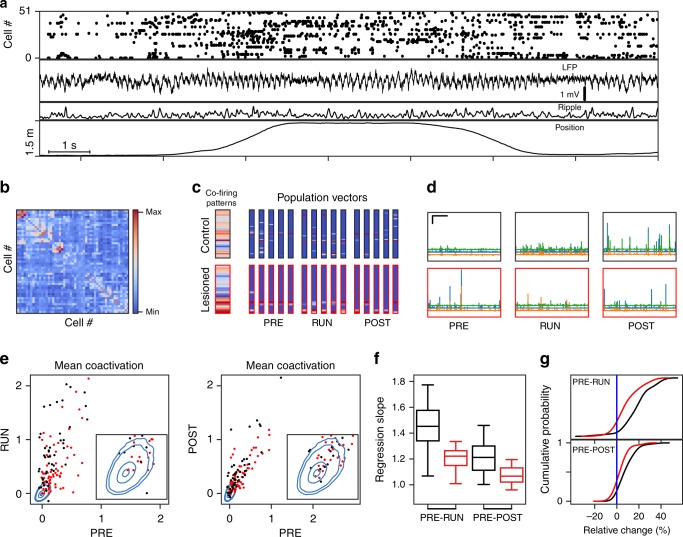
Pattern co-activation. **a** Example of recorded hippocampal activity from a control animal during a RUN session. From top to bottom: Raster of single unit-activity of 51 cells, local field potential, ripple power (in a.u.), and position on linear track. **b** Covariance matrix obtained from *z*-scored population rate vectors within theta cycles throughout the RUN session from A. **c** Two examples of significant eigenvectors (wide columns) from the covariance matrix and five matching example pattern vectors from the PRE, RUN, and POST rest sessions. Red frames indicate examples from a lesioned animal. Colors as in **b**. **d** Example of three co-activation traces (scalar product between patterns and eigenvectors) as a function of time during PRE and POST rest epochs (scale bars: horizontal 100 s, vertical 50 standard deviations, traces are offset parallel to *y*-axis for visual distinction). **e** Session-wise mean RUN (left) and POST (right) co-activation traces vs. mean PRE co-activation traces for all significant eigenvectors from control animals (black, Ctr.) and MEC-lesioned animals (red, Les.). The blue contours (1, 5, 50, and 90%) delineate isoclines of the null distribution obtained from 50,000 random permutations of cell indices. **f** Session-wise regression slopes of PRE vs. RUN and PRE vs. POST. Whiskers represent 1.5 interquartile range. Comparison between animal groups; PRE–POST ranksum test: *p* = 0.00057, r.s. = 225, *n* = 29 slopes; PRE–RUN ranksum test: *p* = 0.0011, r.s. = 221, *n* = 29 slopes. **g** Cumulative histogram of relative change [top: (POST–PRE)/(POST + PRE); bottom: (RUN-PRE)/(RUN + PRE)]. Signed-rank tests show PRE–POST control: *p* = 2.4E-8, s.r. = 60, *n* = 50 patterns; MEC-lesion *p* = 1.6E−4, s.r. = 642, *n* = 72 patterns; PRE–RUN control: *p* = 1.98E−8, s.r. = 56.0, *n* = 50 patterns; MEC-lesion *p* = 1.59E−7, s.r. = 380, *n* = 72 patterns. Comparison between PRE–RUN and PRE–POST: Control: *p* = 2.4E−06, s.r. = 149, *n* = 50 patterns, MEC-lesion: *p* = 1.0E−4, s.r. = 623, *n* = 72 patterns; signed-rank test. Comparison between animal groups; PRE–POST ranksum test: *p* = 3.2E−4, r.s. = 3766; PRE–RUN ranksum test: *p* = 4.98E−6, r.s. = 3952

To compare the sequential activation of hippocampal cells between sharp wave and theta states, we performed extracellular recordings from the hippocampal CA1 region during a new spatial experience as well as during rest epochs in a familiar box before and after the new experience. The two rest epochs are referred to as PRE and POST, respectively. The new spatial experience consisted of running back and forth between two reward locations on a linear track with the track placed in either a new room or in a room with a completely new set of visual cues. The behavior is subsequently referred to as RUN epoch. As expected, neuronal activity and field potentials recorded during RUN epochs showed theta oscillations during movement and sharp wave ripples during immobility while consuming the reward (Fig. 1a). For analysis of replay, templates were generated from the movement periods in RUN, and similarity to these templates was then evaluated for PRE and POST as well as for immobility within RUN. All measurements were compared between control and MEC-lesioned rats (see Supplementary Fig. 1 for lesion extent). In some rats, the MEC-lesions also extended to the parasubiculum, which apart from a possible polysynaptic thalamic connection to the hippocampus^35^, projects strongly via layer II of the entorhinal cortex to the hippocampus^36,37^. Based on these connection patterns, we suggest that MEC-lesions with and without parasubicular damage deprive the hippocampus not only from medial entorhinal inputs but also from the most direct parasubicular input route to the hippocampus and thus did not expect additional effects that were related to the extent of damage to the parasubiculum.

Because it has been reported that place fields are observed in a smaller proportion of hippocampal cells in MEC-lesioned rats compared to controls^34^, we validated that all analyses were either robust to these differences or controlled for these differences. We first used an analysis of co-activation patterns that did not require the delineation of place fields and rather made use of all active cells during locomotion in RUN (see Methods and ref. ^38^) to define orthogonal template patterns. Using the theta periods in RUN epochs, we generated a population rate vector for each theta cycle of the local field potential (LFP) and computed the covariance matrix of these vectors after cell-wise normalization (Fig. 1b). Subsequently, recurring common co-activation patterns were identified by calculating the significant principal components (PCs) of this matrix (Fig. 1c). Projections of the population rate vectors over the time course of each of the three rest/immobility epochs (PRE, immobility-RUN, and POST) to the PCs from locomotion periods in RUN (Fig. 1d) then yielded a co-activation trace for each epoch and PC. Co-activation events were identified as the peaks of the traces throughout the time course of each epoch (event rates are quantified in Supplementary Note 1).

Mean co-activation strengths for each template pattern were then obtained by averaging the corresponding co-activation trace over the entire time within the epoch. Pattern-wise mean co-activation strengths were significantly above chance in PRE, Immobility-RUN, and POST epochs for both control and MEC-lesioned animals (ranksum test of co-activation values *z*-scored relative to surrogate; *p* = 2.7E−18, r.s. = 5,778,263, *n* = 50 patterns, for PRE control; *p* = 4.2E−28, r.s. = 6,460,088, *n* = 50 patterns, for RUN control; *p* = 6.07E−27, r.s. = 6,336,366, *n* = 50 patterns, for POST control; *p* = 1.87E−36, r.s. = 9,030,239, *n* = 72 patterns, for PRE MEC-lesion; *p* = 2.6E−41, r.s. = 9,311,741, *n* = 72 patterns, for RUN MEC-lesion; *p* = 4.3E−40, r.s. = 9,242,817, *n* = 72 patterns, for POST MEC-lesion; see Fig. 1e). We next tested the similarity of the co-activation patterns between different epochs by calculating the mean co-activation strength for each pattern in each epoch and by correlating the co-activation strengths between epochs. Significant correlations were obtained between PRE and RUN as well as between PRE and POST in both groups (Fig. 1e; PRE–POST control: Spearman’s *r* = 0.73, *p* = 7.3E−10, *n* = 52 patterns; MEC-lesioned: *r* = 0.77, *p* = 0, *n* = 72 patterns; PRE–RUN control: Spearman’s *r* = 0.7, *p* = 5.7E−9, *n* = 52 patterns; MEC-lesioned: *r* = 0.5, *p* = 7.6E−6, *n* = 72 patterns). The similarity of all subsequent epochs to PRE indicates that the RUN epoch had only a limited effect on occurrence of the patterns of co-active neurons that persisted in POST after the RUN epoch. This finding is in line with the idea of pre-existing activity schemas that are utilized during novel experiences and also played out afterward.

Although we found that the strength of individual co-activity patterns correlated between epochs, we reasoned that the overall magnitude of co-activation may nonetheless be higher in a subset of epochs. To identify whether the co-activation strength of the later epochs (i.e., RUN and POST) deviated from PRE, we calculated the regression slopes between the strength of PRE and RUN and between the strength of PRE and POST co-activation patterns (Fig. 1f). The observed slopes were significantly larger than the slopes derived from the surrogate data in Fig. 1e (Control: PRE–POST and PRE–RUN 10 out of 10 sessions were significant, *p* = 0 binomial test; MEC-lesioned PRE–POST 10 out of 19 sessions were significant, *p* = 2.5E−10; PRE-RUN: Out of 19, 18 sessions were significant, *p* = 2.0E−25; see Table 1) which is an indication that pre-existing patterns were amplified by the RUN epoch, even in the MEC-lesion group. Accordingly, the relative change in co-activation was significantly positive in both groups of animals (Fig. 1g; see legend for statistics) Although patterns were strengthened in both groups, the MEC-lesion group showed a significantly lower amplification of the co-activation by the RUN epoch compared to the control groups (comparison between regression slopes from see Fig. 1f legend). Taken together, these results indicate that the boost of pattern activation by behavior is highest within RUN and decays from RUN to POST epochs (Fig. 1g; see legend for statistics) and is reduced but not abolished by MEC-lesions. To ensure that our results are not dominated by single animals, we confirmed our analysis by only considering patterns from one session per animal (with most units; Supplementary Fig. 6a–c, Supplementary Table 4).

**Table 1:** Amplification of co-activation strengths as measured by regression slope

Control				MEC-lesioned			
PRE–RUN		PRE–POST		PRE–RUN		PRE–POST	
Slope	*p* Value	Slope	*p* Value	Slope	*p* Value	Slope	*p* Value
1.37	<2e−5	1.21	<2e−5	1.45	<2e−5	1.07	0.006
1.77	<2e−5	1.20	<2e−5	1.20	<2e−5	*1.03*	*0.1*
1.67	<2e−5	1.06	0.003	1.10	4 e−3	*1.03*	*0.12*
1.52	<2e−5	1.28	<2e−5	*1.00*	*0.4*	1.06	0.01
1.45	<2e−5	1.11	2e−4	1.08	0.002	*0.99*	*0.6*
1.44	<2e−5	1.23	<2e−5	1.37	<2e−5	1.06	0.02
1.24	<2e−5	1.33	<2e−5	1.20	<2e−5	1.16	<2e−5
1.43	<2e−5	1.25	<2e−5	1.12	1e−4	*0.82*	*1.0*
1.66	<2e−5	1.22	<2e−5	1.23	<2e−5	1.19	<2e−5
1.54	<2e−5	1.16	<2e−5	1.22	<2e−5	1.18	<2e−5
				1.08	0.003	*0.96*	*0.92*
				1.64	<2e−5	1.12	<2e−5
				1.31	<2e−5	*0.97*	*0.84*
				1.28	<2e−5	1.13	4e−5
				1.10	7e−4	*1.02*	*0.16*
				1.70	<2e−5	*1.02*	*0.27*
				1.20	<2e−5	1.1	5e−4
				1.09	6e−4	*1.02*	*0.15*
				1.13	<2e−5	1.09	2e−3

Regression slopes from Fig. 1e for all 10 sessions (rows) analyzed from control animals and all 19 sessions analyzed from MEC-lesioned animals. *p* Values are obtained from the distribution of regression slopes obtained by 50,000 shuffles of cell indices (blue distributions in Fig. 1e), where the regression line was fitted to as many data points as there were patterns in the real data of the specific session. Italic numbers indicate nonsignificant sessions

### Effects of MEC-lesions on sequence replay

**Fig. 2:**
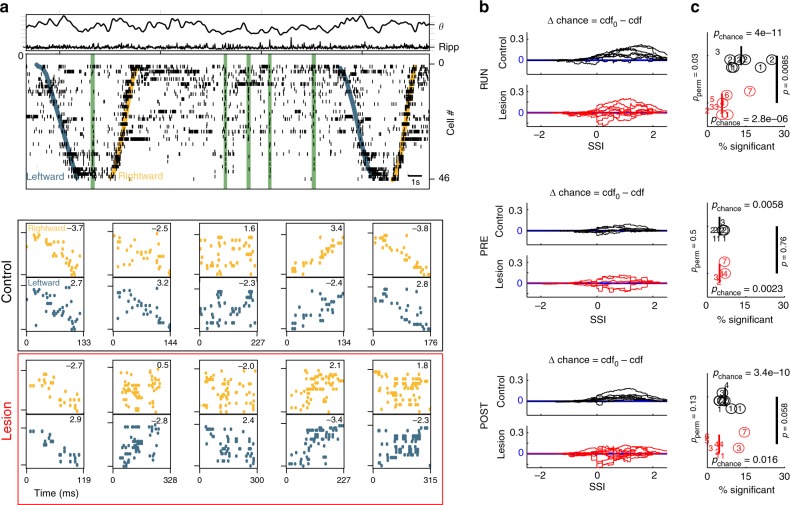
Spatial sequences. **a** Top: Example data from a RUN epoch of a control animal. Top: LFP theta and ripple power (in a.u.). Bottom: Spike raster plot where cells are ordered according to their place field centers on rightward runs (yellow) and leftward runs (blue). Green bars indicate place cell bursts. Bottom: Examples of zoomed in place cell bursts for rightward (top, spikes in yellow) and leftward (bottom, spikes in blue) runs. Numbers are respective SSIs. Black box: examples from the control group; Red box: examples from the MEC-lesion group. **b** Excess cumulative SSI probabilities [difference between cumulative SSI distributions from the shuffled data (cdf0) and the actual data (cdf)] for all sessions in control (black) and MEC-lesioned (red) animals under the three recording conditions (RUN, PRE, and POST). Positive cdf0–cdf indicates a rightward shift of cdf, i.e., larger real than shuffled SSIs. **c** Epoch-wise percentages of significant SSIs. Each epoch is depicted by one numeric symbol. Different numbers encode animal identity (control: 1–4, MEC-lesion: 1–7). Numbers in circle indicate significant epochs. *p*_chance_: *p* value of binomial test on the number of significant sessions given the expectation from the criterion of 5%, *p*: *p* value of ranksum test comparing the medians of animal groups, RUN: r.s. = 105, PRE: r.s. = 57, POST: r.s. = 120; PRE: *n*_control_ = 8 epochs, *n*_lesion_ = 6; RUN: *n*_control_ = 8 epochs, *n*_lesion_ = 10; POST: *n*_control_ = 9 epochs, *n*_lesion_ = 11. The *p* values *p*_perm_ on the left are derived from a comparison between the animal groups based on a permutation test in which we kept all epochs from one animal together while shuffling group labels of the animals (Supplementary Note 1). Based on this procedure we can rule out that the group differences are only due to single animals

The analysis of the mean co-activation strength that we have described so far represents a compound signal that measures the similarity of orthogonal patterns during theta states to population bursts as well as the rate of burst events that show similarity. Furthermore, the measurement considers co-activation of all neurons and does not specifically take into account the temporal order of place cell spikes. We therefore next asked whether the observed amplification of co-activation in immobility-RUN goes along with an increase in sequence replay. For the analysis of replay, we considered units which showed clear place fields in at least one running direction on the linear track (*n* = 414 place cells in controls; *n* = 247 place cells in lesioned animals; Supplementary Fig. 2a). From these cells we constructed two template sequences, one for each running direction (Supplementary Fig. 2b). We then extracted spike sequences from place cell bursts (consisting of at least five place cells during periods when the population activity exceeded three standard deviations over the mean) during PRE, RUN, and POST epochs (Fig. 2a); event rates are quantified in Supplementary Note 2. To account for the different numbers of place cells in different sessions, we analyzed sequences by using scaled rank order correlation coefficients (see Methods). Briefly, the rank order correlation coefficient of cell indices was normalized by the standard deviations of sequence lengths distributions (see Methods; Fig. 2a bottom) to compute a spatial similarity index (SSI). By including the *z*-score normalization, we accounted for the effect that the distributions of correlation coefficients in the surrogate data were increasingly concentrated at zero with longer sequence length (Supplementary Fig. 2c, d). With the normalization, we were able to compare between sessions with different numbers of place cells. The independence of the analysis from the number of recorded cells was further confirmed by repeating the SSI analysis with down-sampled place cell numbers (see below). The SSI thus represents a length-independent measure of how well an activity sequence from a place cell burst matches a template sequence.

After computing SSIs from the real data, the SSIs were compared to those derived from random permutations of the cell indices from the sequences in place cell bursts recorded during PRE, immobility-RUN, and POST periods. The resulting cumulative excess probability (Δ chance) of SSIs during all three types of epochs (PRE, RUN, and POST) in both animal groups are shown in Fig. 2b. Δ chance in all cases exhibited a positive bias for larger SSIs in real than in shuffled data, indicating that sequences tend to be more similar to spatial templates than chance. To assess the statistical significance of this bias, we computed the fraction of replays that exceeded the epoch-wise 95% quantile of the SSI distribution obtained from index permutations (Fig. 2c) and did a binomial test on whether the number of significant epochs (animal numbers with circles in Fig. 2c) exceeded the chance level of 5%. The fraction of significant epochs was significantly above chance for all epochs in both MEC-lesioned and control groups. In at least a small fraction of sessions, spike sequences during rest/immobility periods were thus correlated to the spatial templates (circles in Fig. 2c indicate 7 significant epochs out of 8 during RUN control, 5 out of 10 during RUN lesion, 2 out of 8 during PRE control, 2 out of 6 during PRE lesion, 7 out of 9 during POST control, 2 out of 11 during POST lesion).

To compare the level of replay between control and MEC-lesioned animals we examined the differences of the fractions of significant replay sequences using ranksum tests (Fig. 2c, *p* values as indicated near the black bars in Fig. 2c, ranksum values are provided in the Figure caption). We found a significantly larger percentage of significant sequences in control compared to MEC-lesioned animals for RUN epochs. For PRE and POST epochs the ranksum test on the medians (Fig. 2c) showed no significant differences between the control and MEC-lesion group. Because these results suggest that control but not MEC-lesioned animals show particularly high replay in the RUN epochs, we also compared fractions of events with significant replay across time (PRE, RUN, and POST) in a single session and indeed found the fractions to be significantly higher for immobility-RUN epochs compared to PRE and compared to POST epochs in control animals (PRE vs. RUN; *p* = 0.0079, s.r. = 1, *n* = 8 epochs; POST vs. RUN; *p* = 0.020, s.r. = 33, *n* = 8 epochs; one-sided signed-rank test) but not in MEC-lesioned animals (PRE vs. RUN; *p* = 0.35, s.r. = 8, *n* = 6 epochs; POST vs. RUN; *p* = 0.29, s.r. = 28, *n* = 9 epochs; one-sided signed-rank test). Replay analysis was also performed per animal (Supplementary Note 3) and using a downsampling approach to equalize burst numbers between sessions (Supplementary Note 4), and both methods yielded results consistent with the full dataset.

Interestingly, the results from the SSI analysis are generally consistent with our findings from the co-activation analysis, with both types of analyses showing a strong boost of replay in RUN epochs of control animals, but weaker amplification in MEC-lesioned animals. However, the rate of significant place cell sequence events (Supplementary Note 2) did not show a significant correlation with the rate of pattern co-activation (Spearman’s *r* = 0.14, *p* = 0.43, *n* = 34 epochs), and the coincidence probabilities were low (Supplementary Table 3) indicating that co-activation patterns and place cell sequences can be independently amplified by run sessions. Differences between the two analysis methods (e.g., in rates of bursts vs. rates of patterns) thus likely result from including all cells on the co-activation analysis vs. only including identified place cells in the sequence analysis.

**Fig. 3:**
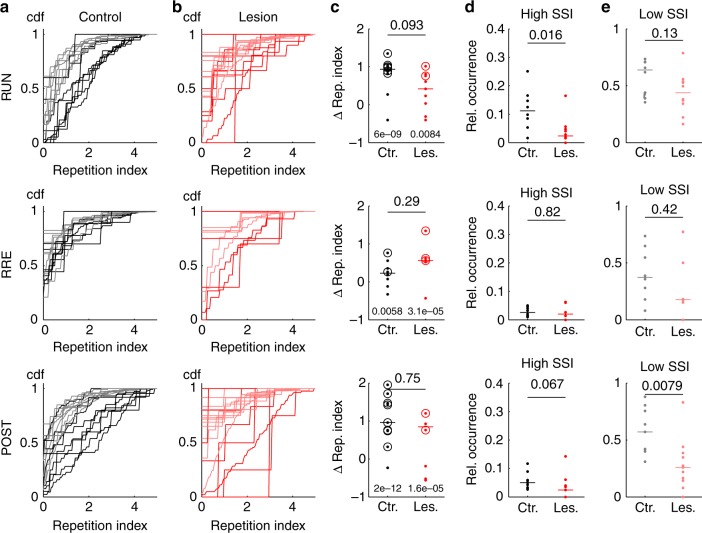
Spatial sequences recurred more frequently than nonspatial sequence motifs. **a** CDFs of repetition indices for spatial (significant SSI from Fig. 2b, c; black) and significant sequence motifs with low SSI (gray) in control animals for epoch types (RUN: *n*_control_ = 7 epochs; PRE: *n*_control_ = 7 epochs; POST: *n*_control_ = 9 epochs) as indicated. Each CDF is derived from one epoch. **b** Same as in (**a**) for MEC-lesioned animals (red: significant SSI, pink: nonsignificant SSI; RUN: *n*_lesion_ = 9 epochs; PRE: *n*_lesion_ = 5 epochs; POST: *n*_lesion_ = 8 epochs). **c** Difference of medians between high- and low-SSI CDFs from (**a**) and (**b**), *p* values above bar were obtained from ranksum test, RUN: r.s. = 90, PRE: r.s. = 48, POST: r.s. = 85; *p* values above group labels were obtained from a binomial test on the number of significant epochs (circles) for a chance level of 5%; RUN: 6 out of 8 epochs were significant in control animals, 2 out of 9 epochs were significant in MEC-lesioned animals; PRE: 2 out of 8 epochs were significant in control animals, 3 out of 5 epochs were significant in MEC-lesioned animals; POST: 8 out of 9 epochs were significant in control animals, 4 out of 8 epochs were significant in MEC-lesioned animals; colors as in (**a**) and (**b**). **d** Relative (Rel.) occurrence computed as the fraction of spatially significant sequences among all bursts (*p* value obtained from ranksum test, RUN: r.s. = 103, PRE: r.s. = 62, POST: r.s. = 119; colors as in (**a**) and (**b**). **e** Same as **d** for low-SSI motifs (RUN: r.s. = 94, PRE: r.s. = 67, POST: r.s. = 130)

In order to further examine how MEC-lesions affect hippocampal spike-timing, we analyzed recurring activity motifs that do not correspond to spatial templates, but may nonetheless contribute to pattern activation^39^. To identify such motifs, we repeated a similar analysis as for the spatial templates, but this time computed a motif similarity index (MSI) as normalized rank order correlation coefficient between all pairs of sequences during one epoch in a recording session. The ensuing analysis is summarized in Supplementary Fig. 3 and yielded that under all conditions, the number of epochs where motifs were detectable significantly exceeded chance level. Motifs were similar between PRE and POST epochs, and this similarity was not different between control and lesion group. Thus, the hippocampal network expressed stable temporal patterns that are not affected by behavior. We did, however, again not find overall correlations between significant motif rates and pattern activation rates from the co-activation analysis (Spearman’s *r* = 0.22, *p* = 0.20, *n* = 35 epochs), suggesting that significant motifs are not the major factor in explaining co-activation patterns and that co-activation patterns are likely also influenced by nonsequence type activity. To quantify how much the significant motifs relate to significant spatial sequences, we counted how many observed sequence motifs were similar (MSI in the upper 5% quantile) to a significant spatial sequence. For quantification, we computed a repetition index for each of the sequences, which is the number of its repetitions (corresponding to significant MSIs) normalized to account for differences in population bursts per epoch (see Methods). The cumulative distributions of repetition indices (Fig. 3a, b) show that significant spatial sequences were generally repeated more often than other significant motifs. For all epochs in the control and MEC-lesion group, the number of epochs in which the repetition index of spatial sequences exceeded that of motifs was significantly larger than expected by chance (*p* values from binomial tests provided in Fig. 3c). The differences between control and MEC-lesion groups did not reach significance (*p* values from ranksum tests above bars in Fig. 3c). Although sequences with high spatial similarity were generally replayed more frequently than motifs (Fig. 3c), they only made up a small fraction of the overall number of population bursts (Fig. 3d). This fraction was larger in control animals than in MEC-lesioned animals only during RUN (ranksum test; *p* values and ranksums as indicated in Fig. 3d and legend), again reflecting the more pronounced amplification of behavior-related sequences during RUN in controls. Furthermore, the data also indicate that burst activity includes a large pool of sequence motifs (Fig. 3e) that are unrelated to the sequences during the behavioral task on the recording day. Again animal-wise analysis showed that our results from session-wise motif analysis are not dominated by outliers (Supplementary Fig. 6e).

**Fig. 4:**
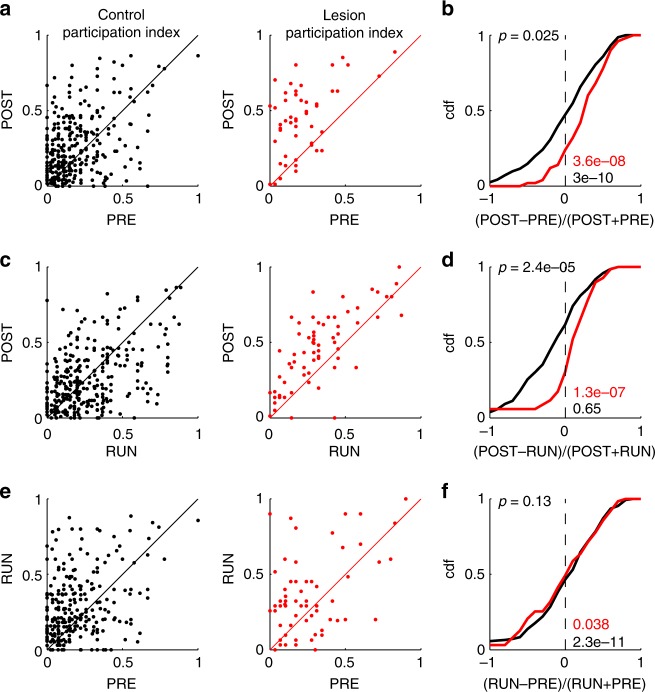
Place cell participation in spatial sequences. **a**, **c**, **e** Correlation of cell-wise participation in significant spatial sequences between two epochs. Each dot indicates the participation of a place cell in significant spatial sequences in the two epochs indicated on the axes (POST vs. PRE: **a**, **b** POST vs. RUN: **c**, **d**; RUN vs. PRE: **e**, **f**). Left: control animals (black); right MEC-lesioned animals (red). **b**, **d**, **f** CDFs of the relative changes (as indicated at the *x*-axis) from the data in (**a**, **c**, **e**). The *p* values at the bottom right were derived from signed-rank tests of medians larger than 0 (PRE–POST Control: s.r. = 33274, *n* = 312 place cells; PRE–POST MEC-lesioned: s.r. = 1281, *n* = 52 place cells; RUN–POST Control: s.r. = 23794, *n* = 314 place cells; RUN–POST MEC-lesioned: s.r. = 2290, *n* = 73 place cells; PRE–RUN Control: s.r. = 34498, *n* = 310 place cells; PRE–RUN MEC-lesioned: s.r. = 1344.5, *n* = 65 place cells). The *p* value on the top left was obtained by a ranksum test on identical group medians (PRE–POST: r.s. = 55,364, *n*_control_ = 312 place cells, *n*_lesion_ = 52 place cells, RUN–POST: r.s. = 57,277, *n*_control_ = 314 place cells, *n*_lesion_ = 73 place cells; PRE–RUN: r.s. = 59,490, *n*_control_ = 310 place cells, *n*_lesion_ = 65 place cells)

To examine whether there are further qualitative differences between the sequence replay in control animals and MEC-lesioned animals, we analyzed the contribution of individual place cells to sequences by counting how often a single place cell was active in a significant spatial sequence. The count was then normalized by the total number of significant sequences in an epoch to yield a participation index. The participation index revealed an increase in the participation of single place cells in significant sequences from PRE to POST (Fig. 4a, b) and from PRE to immobility-RUN (Fig. 4e, f) in both control and lesioned animals. However, the magnitude of participation across epochs showed a striking difference between control and MEC-lesioned animals when considering the changes between immobility-RUN and POST. While in control animals there is no significant increase of participation indices of place cells on average (Fig. 4c, d), participation indices in MEC-lesioned animals are almost exclusively increased (Fig. 4c, d). The differential effect of MEC-lesions on participation indices thus seemed to be largely expressed during the POST epoch (Fig. 4b, d, f). This finding suggests that the limited degree of persisting replay in POST epochs of lesioned animals is mostly supported by an increased recruitment of place cells that have already been active in these sequences during the PRE epoch.

### Replay and co-activation during high-frequency events

**Fig. 5:**
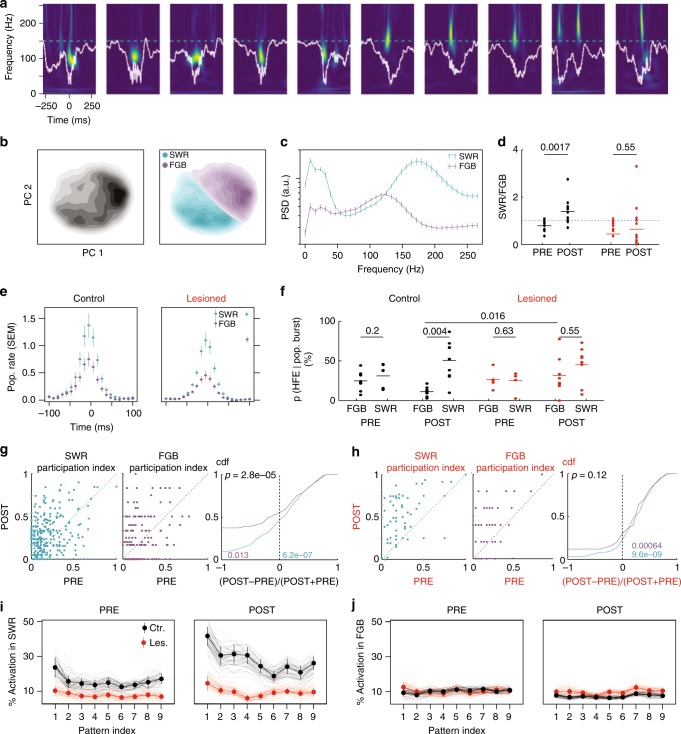
Local field potential correlates of pattern activation and place cell bursts. **a** Ten example traces (white) and corresponding power spectrograms of HFEs (color scale normalized; warm colors indicate high power). Dashed cyan line indicates 150 Hz. **b** Left: Density plot of principal component (PC) projections of the spectrogram from one example session (PRE and POST) of a control animal. Right: Same plot with cluster labels. **c** Power spectral densities from (**b**). Error bars: 99th-percentile. **d** Fraction of SWRs vs. FGBs in the PRE and POST epochs (black: Control; red: MEC-lesion, blue dashed line indicates unity; see main text for statistics). **e** Population rates (cell-wise *z*-scored) triggered by the peak of the HFE. Error bars are 68th-percentiles. **f** Percentages of place cell bursts that coincide with an HFE (±150 ms, colors as (**d**)). *p* Values above bars from signed-rank tests (Control PRE: s.r. = 8, *n* = 8; Control POST: s.r. = 0, *n* = 9; MEC-lesioned PRE: s.r. = 5, *n* = 5; MEC-lesioned POST: s.r. = 13, *n* = 8) and a ranksum test when comparing between animal groups (see main text). **g** Cell-wise participation in significant spatial sequences. Only significant replays are considered in a ±150 ms window triggered by an HFE. Right: CDF of relative changes. *p* Value (black) from ranksum test for group medians (r.s. = 83,934, *n*_SWR_ = 306 place cells, *n*_FGB_ = 198 place cells). Colored *p* values from signed-rank tests for median being negative (purple, FGB; s.r. = 7180, *n* = 198 place cells) or positive (cyan, SWR; s.r. = 29305, *n* = 306 place cells). **h** Same as (**g**) for MEC-lesioned animals (ranksum test comparing FGB and SWR: r.s. = 4573, *n*_SWR_ = 70, *n*_FGB_ = 51; purple *p* value from signed-rank tests for positive median, s.r. = 1007, *n* = 51; cyan: s.r. = 1985, *n* = 70). **i** Percentage of pattern activation events coinciding with an SWR (±50 ms). Labels on the *x*-axis indicate the pattern (principal component) from the co-activation analysis (see main text for statistics). Error bars indicate 66th-percentiles. **j** Same as (**i**) for FGBs

Replay events have been shown to correlate with sharp wave ripple events (SWRs), however, not all population bursts are accompanied by SWRs^3^. Given that fractions of significant replays vary substantially across sessions (Fig. 2c), we were asking whether SSIs and SWRs are related. To this end, we performed a spectral analysis of field recordings to identify SWRs. The peaks in the ripple band (100–250 Hz) were selected as candidates for high-frequency events (HFE) and the power spectra (Fig. 5a) of each event were then subjected to PC analysis (Fig. 5b). The first two PCs allowed us to generally identify two clusters of HFEs, one with a clear high-frequency peak at 150–200 Hz, called SWR in the following, and another one with a spectral peak between 100 and 150 Hz, which we called fast gamma burst (FGB) (Fig. 5c; see Supplementary Fig. 4 for all sessions; cf. ^40^). The ratio of SWRs to FGBs in control animals was significantly increased in POST epochs (*p* = 0.0017, r.s. = 78, *n*_PRE_ = 11 epochs, *n*_POST_ = 11 epochs; ranksum test; Fig. 5d). In MEC-lesioned animals, there was no such increase (*p* = 0.55, r.s. = 378, *n*_PRE_ = 20 epochs, *n*_POST_ = 19 epochs; ranksum test), which indicates that at least in control animals the two types of HFEs are functionally distinct between PRE and POST epochs.

To observe whether effects on HFEs also relate to spatial sequences, we examined multiunit spiking activity and found that spiking activity was generally enhanced in SWRs compared to FGBs in both MEC-lesioned and control animals (Fig. 5e). Moreover, in POST epochs, FGBs were more frequently occurring during bursts in the lesioned group than in the control group (*p* = 0.016, r.s. = 56, *n*_control_ = 9 epochs, *n*_lesion_ = 8 epochs, ranksum test; Fig. 5f). This effect led to similar burst fractions in FGBs and SWRs in the lesioned group, whereas SWRs remained more prominently locked to place cell bursts in the control group (*p* = 0.004, s.r. = 0, *n* = 9, epochs; signed-rank test, Fig. 5f; see Supplementary Fig. 6f for animal-wise analysis). To test whether the differential association of HFE type with bursts also carries over to place cell participation (Fig. 5g), we separately computed participation indices for POST epoch SWRs and FGBs in control and MEC-lesioned animals. In the control group, participation was significantly increased during SWRs of the POST epoch (as compared to participation in all significant sequences of the PRE epoch) whereas participation was significantly decreased during FGBs (Fig. 5g; signed-rank tests; *p* values as indicated in colors) leading to a significantly larger participation during POST SWRs than during POST FGBs (ranksum test on relative changes as indicated in Fig. 5g). In the lesion group, we observed significant increases in the participation index during both SWRs and FGBs (Fig. 5h). The elevated rather than decreased participation during FGBs in MEC-lesioned animals thus appears to explain the increased participation during POST (see Fig. 4).

Finally, to connect the findings on the distinct patterns of HFEs in the control and lesion animals to the co-activation analysis (see Fig. 1), we computed how many co-activation events coincided with HFEs. In control animals, we found that co-activation events were only enhanced from PRE to POST epochs when they coincided with SWRs (*p* = 3.1E−9, *T* = −6.13, *n* = 270 patterns, *t* test; Fig. 5i) and they were even slightly lower during FGBs (*p* = 4.5E−6, *T* = 4.68, *n* = 268 patterns, *t* test; Fig. 5j). These results corroborate the differential role of the two types of HFEs in that an enhancement of activation occurs during SWRs, whereas a reduction is associated with FGBs. MEC-lesions seem to disrupt both of these processes. In lesioned animals, the coincidence of co-activation and HFEs did not differ between PRE and POST (SWR: *p* = 0.1, *T* = −1.6, *n* = 327 patterns; FGB: *p* = 0.53, *T* = 0.62, *n* = 333 patterns; *t* test) and SWR-associated co-activation was lower than in control animals during both epochs (PRE: *p* = 7.9E−10, *T* = 6.3, *n* = 302 patterns, POST: *p* = 1.1E−21, *T* = 10.37, *n* = 295 patterns; *t* test; Fig. 5i). During POST, but not PRE epochs, the co-activation during FGBs was even significantly larger than in control animals (POST *p* = 3.9E−4, *T* = −3.59, *n* = 296 patterns; PRE: *p* = 0.86, *T* = −0.17, *n* = 305 patterns; t test). Thus, the facilitation of co-activation during SWRs and the decrease of both co-activation and place field participation during FGBs was MEC-dependent.

## Discussion

Hippocampal pattern activation and sequence replay were examined in animals with extensive bilateral lesions of the MEC. Despite a strong reduction of hippocampal phase precession and strongly disrupted spike-timing correlations with these lesions^29^, we observed almost control-level pattern co-activation during PRE, RUN, and POST resting periods, and some of the MEC-lesioned animals exhibited retained sequence replay at control levels, in particular during PRE and POST epochs. In addition, we found a strong intrinsic network structure prior and subsequent to the behavioral session that may either reflect previous experience or developmental biases. The degree of similarity of the intrinsic patterns did not differ between control and MEC-lesioned animals. Although many aspects of replay were thus retained in lesioned animals, we also observed that the enhancement of co-activation and sequence replay that is observed during immobility in RUN and POST epochs in controls is diminished without MEC inputs to the hippocampus. The temporal organization during theta states that is provided by MEC inputs therefore appears to facilitate behavior-related plasticity of population activity, but to not disrupt the retrieval of pre-existing functional connectivity. Thus, the hippocampal network is more rigid without the precise spike-timing during theta states.

Transitions from down to up states in the entorhinal cortex are a major trigger of hippocampal sharp waves^40–43^. The population bursts we observed in MEC-lesioned rats may therefore either be caused by down up transitions in the lateral entorhinal cortex^44^ or be generated in the hippocampus intrinsically^45^. Further evidence that the isolated hippocampus can generate sharp waves was reported in refs. ^46,47^, who even found overall increased numbers of sharp waves after animals recovered from bilateral MEC-lesions. Conversely, acute optogenetic inactivations of MEC layer III during quiet wakefulness found sharp wave ripples at a reduced incidence rate^48^. Our analysis of resting period activity did not show increased or decreased rates of either place cell bursts or pattern co-activation rates in lesioned animals.

In addition to the reduced rate of SWRs, MEC layer III inactivation has also been reported to result in restricted sequence replay of place field sequences of familiar linear tracks^48^ indicating a specific role of MEC inputs in supporting specifically long sequences, which we cannot address with the limited number of place cells from our MEC-lesion group. Our results from chronically lesioned animals show that the MEC also has specific effects on hippocampal CA1 activity sequences related to novel experiences. These are not only reflected through changes in the relative occurrence of behaviorally related activity patterns (Figs. 1 and 2), but also through an altered plasticity of significant spatial sequences. Participation of already existing place cells during POST epochs was mainly increased in MEC-lesioned animals, whereas in control animals participation of individual place cells exhibited cell-specific increases and decreases (Fig. 4). The failure to reduce participation during the POST epoch in some of the MEC-lesioned animals might reflect that schemas^16^ that the animals has acquired prior to the lesion have become more prominent and tend to reverberate in the hippocampus. The acquisition of these schemas has presumably occurred by synaptic plasticity already prior to the lesion when place cell correlations were still intact^29^.

While HFEs have in general been shown to correlate with pattern activation (e.g., ^3,6,9^; see ref. ^49^ for review), our data adds that SWRs selectively—as compared to FGBs—boost pattern activation in control animals. Together with the finding that, during FGBs, place cells of MEC-lesioned animals that were active during PRE do not exhibit decreased participation during POST, our data suggest that, after MEC-lesions, the reactivation of schema-related sequences in FGBs may be incorrectly favored over the replay of information about the new spatial experience (Fig. 5i). While the differential effect of SWRs and FGBs on place cell participation establishes an interesting functional correlate of LFP signals, the causal mechanistic link, however, remains speculative. It is conceivable that distinct plasticity-induced participation rates of the place cell bursts render different field potential shapes, e.g., by differential activity-dependent recruitment of interneuron circuits^50–54^. Alternatively, the distinct types of HFEs might be triggered by differential pathways that then recruit different subsets of place cells leading to distinct participation rates. Because no obvious differences have been found by ref. ^40^ concerning the anatomical pathways triggering of SWRs and FGBs, we currently assume that both types of HFEs may be elicited in the hippocampus in the same way, e.g., via ramping CA2 activity^55^, and that the distinct HFE types result from a differential intrinsic recruitment of cells.

The idea that the recall of a memory trace renders it unstable and allows for its modification by new experiences (Fig. 4c) is well-known in the field of memory reconsolidation^56–58^. For example, Moncada and Viola^59^ could translate short-term into long-term memory by exposing the animal to a novel environment shortly before or after the acquisition of the short-term memory consistent with the synaptic tagging hypothesis^60–62^. The plasticity of place field participation indices that we observed during POST epochs in control animals (Fig. 4) points in a similar direction by revealing that many cells decrease their participation. Animals with MEC-lesions putatively have reduced synaptic plasticity as suggested by the reduced decrease of participation indices (Figs. 4c and 5i). Their memory traces should therefore be less affected by recall. This prediction is consistent with behavioral data from Hales et al.^34^, showing that animals with MEC-lesions exhibit an increased perseverance during reversal learning. The presence of recurring pattern activation (Fig. 1), even in PRE epochs^63^, also fits to expected mechanisms for reconsolidation because the observed increase of mean pattern activation in POST epochs (particularly of control animals), together with the strong correlation between PRE and POST co-activation (Fig. 1d, e), would be predicted if pre-existing patterns were modified during RUN. The smaller POST co-activation in animals with MEC-lesions (Fig. 1g) again suggests that in these animals the pre-existing memory traces are less vulnerable, presumably because the spike-timing correlations are corrupted.

Taken together, a limited amount of behaviorally induced plasticity appears to act on top of a pre-existing network structure during replay. The behaviorally induced plasticity component is disrupted by the disorganized spike-timing in theta states of MEC-lesioned rats. Temporal organization during theta states therefore appears to facilitate behavior-related plasticity of population activity, but seems unnecessary for the activation of pre-existing sequences and patterns. Our data thus reveals features that are predicted by both of the antagonistically discussed classes of models for replay—those that suggest pre-wiring and those that suggest behavior-induced plasticity—and suggests that a framework in which existing schemas are updated by novel experiences best captures the complexity of replay events.

## Methods

### Subjects

The subjects were 11 experimentally naive, male Long–Evans rats weighing between 300 and 350 g at the time of the first surgery. The rats were housed individually on a reversed 12 h light/dark cycle and were randomly assigned to two groups—an experimental group with nearly complete NMDA lesions of the MEC (*n* = 7 animals) and a control group that underwent the same initial surgical procedures but without puncturing the dura or lowering the syringe needle into the cortex (control; *n* = 4). Animal numbers were chosen to be comparable to those reported in related studies. Recordings from two of the control animals and two of the MEC-lesioned animals were previously included in a study that reported diminished phase precession with MEC-lesions^29^, and recordings on a novel linear track from the same animals are analyzed here. However, the other animals from the previously published cohort were not tested on novel tracks. The majority of the data in the present study is thus from a new set of animals, for which phase precession slopes were reduced by MEC-lesions to a comparable extent as previously reported (Supplementary Fig. 5). All subjects underwent a second surgical procedure to implant a 14-tetrode recording assembly. Rats were housed individually on a reversed 12 h light/dark cycle. Following a 1-week recovery period from surgery, rats were food restricted and maintained at ~90% of their ad libitum weight. Behavioral testing and recording sessions were performed in the dark phase of the light/dark cycle as described below. All surgical and experimental procedures were approved by the Institutional Animal Care and Use Committee at the University of California, San Diego, and conducted at the University of California, San Diego according to National Institutes of Health guidelines. The authors were not blind to the animal group at any stage of the experiment.

### Surgery

All surgery was performed using aseptic procedures. Anesthesia was maintained throughout surgery with isoflurane gas (0.8–2.0% isoflurane delivered in O_2_ at 1 L/min). The animal was positioned in a Kopf stereotaxic instrument, and the incisor bar was adjusted until bregma was level with lambda. The bone overlying the target site was removed using a high-speed drill. After completion of each lesion the animal was allowed to recover from anesthesia on a water-circulating heating pad. The control group underwent the initial surgical procedures, but no lesions were made.

In the experimental group (MEC), excitotoxic lesions were produced by NMDA dissolved in aCSF (Harvard Instruments) to provide a solution with a concentration of 10 mg/ml. NMDA was injected at a rate of 0.1 µl/min using a 10 µl Hamilton (Reno, NV) syringe mounted on a stereotaxic frame and held with a Kopf model 5000 microinjector. The syringe needle was lowered to the target (AP: anterior border of the transverse sinus, ML: ±4.6 mm at an angle of 22° from posterior to anterior, DV: −5.2 mm) and left in place for 1 min before beginning the injection. NMDA was injected into eight sites (DV 5.2, −4.7, −4.2, −3.7, −3.2, −2.7, −2.2, −1.7 mm, total volume 1.04 µl) on each side of the brain and was intended to damage the complete area of MEC. After the injection, the syringe needle was left in place for 1 min to reduce the spread of drug up the needle tract.

The second surgery followed the same protocol used for the lesion procedures but in this case a recording assembly with 14 tetrodes was implanted above the cortex dorsal to hippocampus (AP: 4.0 mm, ML: ±2.6 mm). Tetrodes were constructed by twisting four 17 µm polyimide coated platinum–iridium (90%/10%) wires, and the electrode tips were plated with platinum to reduce the impedances to 200–300 kΩ at 1 kHz.

### Behavioral apparatus

Behavior was conducted on linear tracks (lengths: 100 and 150 cm) located in a novel room. The tracks were covered with black contact paper and were elevated 50 cm above the floor. Chocolate sprinkles were used as rewards at the end of each of the extremes of the track. A camera was mounted at the ceiling above the center of the track and connected to a monitor and DVD recorder to track and record the rats’ performance on the track. The testing rooms contained a number of constant, salient visual cues. During the sleep sessions the animals were placed in a Plexiglas holding chamber (30 cm × 56 cm) located in a familiar room.

### Behavioral tasks

Rats were given at least 4 weeks to recover from surgery before the beginning of testing on the linear track. After recovery from surgery, rats were handled and familiarized with the room where the sleep sessions took place. During this period, tetrodes were slowly advanced into the CA1 area of the hippocampus. In addition, rats were allowed to explore the Plexiglas holding chamber for at least twelve 1-h periods before the experiment. Furthermore, 2 out of the 4 control rats and 5 out of the 7 lesioned rats ran an alternation task for an average of 8 days (60 trials per day). All the alternation task sessions took place in the familiar room where the resting box was placed. Together, these experiences ensured a complete familiarization with the resting environment.

During this period, tetrodes were slowly advanced into the CA1 area of the hippocampus. During tetrode advancement and recordings, the signals were preamplified with a unity gain headstage and then recorded with a data acquisition system with 64 digitally programmable differential amplifiers (Neuralynx, Tucson, AZ, USA). Spike waveforms above a threshold of 40–45 µV were time-stamped and digitized at 32 kHz for 1 ms. The rat’s position was tracked at 30 Hz by recording the position of light-emitting diodes that were placed above the head. LFPs were acquired by recording one channel of each tetrode with the filters set to the 1–450 Hz band. As expected^45^, SWRs were not diminished by the MEC-lesion and could therefore be used to guide electrode advancement into the cell layers in all rats.

Recording on the linear track began when tetrodes were stably positioned in the CA1 cell layer. Spikes and LFPs were also recorded while the rat was resting in a transparent holding chamber in a familiar room for 1 h at the beginning and 1 h at the end of each recording day. The room had a light source in a corner at approximately 2 m from the sleep chamber that kept the environment dimly illuminated. After the first sleep period, the animals were transported to a novel room to run back and forth on a linear track for a food reward (chocolate sprinkles). The behavior sessions were 30 min long. Immediately after, the rat was transported back to the familiar room and the second sleep period began. Each animal ran one session per day consisting of three epochs (PRE: sleep1 in familiar box, RUN: linear track in novel environment, POST: sleep2 in familiar box). Behavioral sessions were performed for up to 4 days with each day using a distinct novel environment. For the first 3 days, the novel environments were three different rooms other than the one with the sleep box. If recordings were conducted on a fourth day (in 1 of 4 controls 3 of 7 MEC-lesioned rats), a completely novel set of cues was used in one of the three rooms from one of the previous recording days. Data collection and analysis were not performed blind to the conditions of the experiment.

### Neurohistological methods

At the end of the experimental procedures, rats were administered an overdose of sodium pentobarbital and perfused transcardially with a phosphate buffered solution followed by a 4% paraformaldehyde solution (in 0.1 M phosphate buffer). Brains were then removed from the skull, kept in a solution of 4% paraformaldehyde for 24 h, and transferred to a 30% sucrose solution where they stayed for ~48 h. Sagittal sections (40 µm) were cut with a freezing microtome beginning just lateral to the hippocampus and continuing medially through the hippocampus and MEC. Every section was mounted and stained with cresyl violet to track the hippocampal tetrode locations. Every fourth cresyl violet stained section was used to quantify the MEC-lesion extent with the Cavalieri method^34^. The volume of the spared tissue was estimated for the MEC layer II, MEC layer III, MEC deep layers, dorsal parasubiculum, ventral parasubiculum, and hippocampus. When patches of cells showed signs of disorganization and necrosis, the area was counted as damaged^64^. Sparing in the MEC-lesioned animals is predominantly in the most lateral part of the MEC (Supplementary Fig. 1). Damage to the brain areas other than MEC and parasubiculum was not substantial, as previously reported^34^. None of the rats from the MEC-lesioned group had to be excluded because of the pre-established exclusion criterion of more than 30% tissue sparing in layers II and III.

### General data organization

All analyses were based on population events (co-activation events in Fig. 1, place cell bursts Figs. 2–4, and HFEs in Fig. 5). Some animals contributed data from several experimental sessions that were conducted on separate days with the linear track placed in a distinct novel environment on each day (see labels in Supplementary Fig. 2a). Although some of the cells contributing to population events might thus be the same across days, it is well established that hippocampal place cells completely remap across distinct environments and that co-active cell populations are independent^65^. Population events were thus considered as independent across different sessions from the same animal. For the number of animals used for each group (i.e., *n* = 4 control and *n* = 7 lesioned animals), we obtained least about 1000 place cell bursts in each group for sequence analysis (Supplementary Table 1).

Rest periods during PRE and POST epochs were defined as periods while the animal was placed in a Plexiglas enclosure in a familiar room. Rest periods during RUN epochs were defined when the animal was at the reward site at either end of the track.

### Clustering of firing units

Waveforms of recorded spikes were transformed to PC space, unsupervised clustering was performed on first three PC components using masked EM algorithm implemented in KlustaKwik software^66^. Candidate clusters were evaluated further using MClust software (version 3.5, written by A. David Redish; http://redishlab.neuroscience.umn.edu/Mclust/Mclust.html). Low-quality clusters and clusters suspected of not stemming from a biological sources were excluded from further analysis.

### Place cell identification and burst rates

In each RUN epoch, we computed place maps using 5 cm bins. Place-selective firing rates along with the direction of each run (leftward/rightward) were then put under a two-way ANOVA test in order to find spatially modulated and directional selective units. Those were identified as place cells.

Place cell rates were calculated as the total number of spikes of all place cells per time bin of 1 ms convolved with a Gaussian kernel (*σ* = 30 ms). A pace cell burst was defined as the time span while the place cell rate remained at least one standard deviation (SD) above its average during a period when the peak firing rate reached at least three SD. Only place cell bursts with five or more active cells were considered for the analysis. Burst rates were calculated as number of place cell bursts divided by immobility time in an epoch (PRE, RUN, and POST). For RUN epochs only the time that the animal spent in the reward zone was taken into account. Reward zones were defined individually for each session based on visual judgment of the spatial distribution of running speeds. Epochs with 20 or less place cell bursts were excluded from further analysis.

### Pattern activation analysis

We first excluded rest periods from the RUN epochs from further analysis. The LFP signal of the RUN epochs without immobility periods was Fourier transformed and only the 6–10 Hz band was used for back transformation to obtain the theta wave. The filtered signal was Hilbert-transformed to obtain an estimate of the cycle boundaries. The time spans between two consecutive peaks (Hilbert phase 0) in the filtered signal were considered as theta cycles. The spike sorting procedure identified cells with at least about 1000 spikes that were active in at least one of the experimental epochs (PRE, RUN, and POST). This means that neither possible interneurons nor principal nonplace cells have been excluded from this analysis. In both animals groups the number of place cells was about half this total number of active neurons, and the number of active cells was highly correlated with the number of place cells (Control: *p* = 2.6E−11, *n* = 9 sessions; MEC-lesion: *p* = 3.76E−17, *n* = 17 sessions; linear regression). Firing rates of all such identified units were time binned with one bin per theta cycle and were *z*-scored (removal of cell-wise mean and normalization by cell-wise standard deviation). The covariance matrix was determined from the *z*-scored population vectors in each RUN epoch. Firing patterns were identified as the significant eigenvectors of the covariance matrix. Significance was assumed if the eigenvalue exceeded^38^ a threshold given by the Marcenko–Pastur distribution. Eigenvalues above this threshold cannot be accounted for by random matrices of size (N,N) with N being the number of neurons. An extensive description can be found in ref. ^37^. We checked that the sparsity of the pattern vectors were not different between control and MEC-lesioned animals. The co-activation strengths of significant patterns during rest epochs (PRE/POST) and immobility periods (RUN) were calculated as the inner product of the *z*-scored population rate at each time bin and corresponding firing pattern. Mean epoch-wide co-activation strengths were calculated as time average of individual co-activation strengths of relevant patterns^38^. To assess the rate of co-activation events, we identified local maxima of co-activation strength which were three SDs above their mean.

Numbers of sessions that went into the co-activation analysis are summarized in Supplementary Table 2. To confirm that our results are not biased by including more than one recording session from a subset of animals, we validated our results with analysis that was restricted to only one session per animal (Supplementary Fig. 6a–c).

### Sequence analysis

Activity sequences were defined as the sequence of cell indices ordered with respect to the mean spike time of a cell in a place cell burst. To quantify the similarity of activity sequences we computed rank order correlation coefficients^10,13^ between the index sequence observed in a place cell burst and either the template sequence derived from place field centers (spatial similarity) or the sequence obtained from another population burst (motif similarity). Since the distribution rank order correlation coefficients strongly depend on sequence length (Supplementary Fig. 2c, d), they were normalized by the standard deviation of correlation coefficients obtained from 100,000 random index permutations to yield the respective similarity indices (SSI, MSI). Epochs with less than 20 population bursts were excluded from the analysis (Supplementary Table 1).

For each place cell burst in the epoch under consideration, we constructed surrogate sequences from 100 random shuffles of cell indices. For each of these surrogate sequences, we computed two SSIs one with respect to the leftward and one with respect to the rightward template. The larger of the two SSIs or each surrogate sequence was then used to construct the distribution for the SSI null hypothesis in the specific epoch. The null distribution was thus constructed from 100 times the number of place cell burst surrogate SSIs. We then computed the left and right SSI for each candidate sequence and, if the maximum of the two was within the upper 5% quantile of the Null distribution, the sequence was called significant.

To confirm that our results were not biased by including more than one recording sessions from a subset of animals, we validated our results by pooling the data over all sessions per animal.

### Repetition index

In each epoch, we computed MSIs for all pairs of population bursts. For each population burst in an epoch we counted the number *k* of MSI values that were in the upper 5% quantile of an MSI distribution derived from random permutation of cell indices. This number *k* was divided by the SD of *k* in the respective epoch to yield the repetition index.

### Participation index

For each place cell we counted how often it participated in a significant sequence in one epoch. The participation index was this number divided by the number of significant sequences. Epochs with less than four significant sequences were excluded from the analysis.

### LFP analysis and HFEs

For LFP analysis we included all session from the replay analysis plus additional sessions (Control: 2 sessions, MEC-lesioned: 3 sessions) that had good field potential recordings but too few neurons to qualify for replay analysis.

All recording channels were visually inspected both in time and frequency domains. In each session the least noisy and most stable channels were selected for further analysis. Successively, the selected LFP signal were whitened using a second order autoregressive (AR, 2) model (using python package statsmodels; http://statsmodels.sourceforge.net/), to be able to identify low power spectral features for clustering (see below).

Candidate events were detected using a threshold on the absolute value of Hilbert transform (smoothed using a Gaussian kernel with *σ* = 12 ms) of the band passed (100–250 Hz) LFP. Peaks higher than three SD were recorded as candidate events with event duration defined as times where the absolute value of the Hilbert transform remained above one SD.

Power spectra of the whitened signals of all candidate HFE events were determined using multitaper method resulting in power vectors from 10 to 300 Hz for each event. Power vectors were projected to PC space and clustering was performed on the first two PCs using different clustering algorithms provided in python scikit-learn^67^. Comparing the result of different algorithms (MiniBatchKMeans, SpectralClustering, Ward, Birch) on each dataset we accept the most stable partitioning of data across all clustering methods.

We applied wavelet analysis on each 512 ms window around the peak of an HFE^68^ to ensure that HFEs were isolated in both time and frequency domains.

### Blinding

The authors were not blind to the group identity of the animals while performing the experiments and the analysis.

### Reporting summary

Further information on experimental design is available in the Nature Research Reporting Summary linked to this article.

## Supplementary information

Supplementary Information

Reporting Summary

## Data Availability

The custom MATLAB core routines for sequence analysis are available from the github repository https://github.com/cleibold/ReactivationCode.

## Data availability

The recording data are available upon request to the corresponding author
