# Supplementary material for: Hippocampal CA1 replay becomes less prominent but more rigid without inputs from medial entorhinal cortex
Source: Nat Commun. 2019 Mar 22;10:1341. doi: 10.1038/s41467-019-09280-0 (PMC6430812; doi:10.1038/s41467-019-09280-0)
Supplement: Supplementary file 1 — Supplementary Information [file 41467_2019_9280_MOESM1_ESM.pdf]

Supplementary Material

Hippocampal CA1 replay becomes less prominent but more rigid without inputs from medial entorhinal cortex

Chenani et al.

Supplementary Note 1

The incidence rates of co-activation events were not significantly different between the control and lesion group (Wilcoxon ranksum test; PRE: $p = 0.23$, ranksum (r.s.)= 176, $n_{control} = 10$ epochs, mean $rate_{control} = 0.073$ Hz, n_{lesion}=19 epochs, mean $rate_{lesion} = 0.064$ Hz; POST: $p = 0.67$, r.s.= 141, $n_{control}$=10 epochs, mean $rate_{control} = 0.064$ Hz $n_{lesion} = 19$ epochs, mean $rate_{lesion} = 0.069$ Hz), nor were they different between PRE and POST epochs within either of the groups (Wilcoxon signed rank test; Control: $p = 0.24$, signed rank (s.r.) = 16; Lesioned: $p = 0.49$, s.r.= 78; mean rates and n numbers see above).

Supplementary Note 2

The incidence rates of place cell bursts were lower in MEC-lesioned compared to control rats (ranksum test; PRE: $p = 0.0062$, $n_{control} = 9$ epochs, mean $rate_{control} = 0.20$ Hz, n_{lesion}=12 epochs, mean $rate_{lesion} = 0.047$ Hz; POST: $p = 0.0012$, $n_{control}$=9 epochs, mean $rate_{control} = 0.21$ Hz, $n_{lesion} = 12$ epochs, mean $rate_{lesion} = 0.042$ Hz), which was partly expected because at least 5 active place cells were required for a burst and the number of place cells per session was lower in MEC-lesioned animals (Fig S2a, b, cf. 34). However, even after the place cell numbers were randomly downsampled such that they matched between the control and lesioned group, the difference in burst rates remained (mean +/- SEM, PRE: $rate_{Control}$ = 0.057 +/- 0.023 Hz, $rate_{Lesion}$ = 0.024 +/- 0.009 Hz; ranks um test, $p = 0.030$, r.s. = 130, $n_{Control} = 9$, $n_{Lesion} = 12$; POST: $rate_{Control}$ = 0.054 +/- 0.008 Hz, $rate_{Lesion}$ = 0.021 +/- 0.008 Hz, $p = 0.012$, r.s. = 135, $n_{Control} = 9$, $n_{Lesion} = 12$), which is in contrast to the lack of rate differences between groups for co-activation patterns. This discrepancy already indicates that the two methods rely on different sets of cells and that the recruitment of place cells to population bursts differs between the control and the lesion group.

Supplementary Note 3

Significant replay was also present when pooling data from all sessions per animal (Figure S6d), however, with the smaller number of observations (animals), significant differences in the fractions of significant replay events between animal groups are not attainable with a non-parametric test although a clear trend was observed showing that our session-wise analyses are not dominated by outliers. We therefore also performed a permutation test where we randomly assigned group labels to animals (but kept animal identities for the individual sessions). We then computed test statistics (difference of mean fractions of significant bursts) for 10,000 shuffles from which we could derive the p-value (p_{perm}) for the test statistic obtained with the real group labels. Using this permutation approach the group differences in the RUN sessions remained significant arguing that our results are robust across animals.

Supplementary Note 4

To further confirm that the SSI-based analysis was not biased by the difference in the number of place cells in MEC-lesioned and control animals, we repeated the SSI analysis with data that were downsampled to match the number of recorded place cells between the control and lesion group (Supplementary Figure 2e). Overall, the results from the data before and after downsampling thus further corroborate that behavioral amplification of replay is diminished without MEC inputs.

Supplementary Table 1. Number of observations for sessions and place cell burst under all conditions.

		Control		Lesion	
PRE	Sessions (per animal)	3/4/1/0	**8**	1/1/1/2/0/0/1	**6**
	Place cell bursts (per session)	(299,339,499)/(1768,810,666,563)/262/-	**5206**	69/22/419/72/116/-/-/432	**1130**
RUN	Sessions (per animal)	3/4/1/0	**8**	2/1/2/2/1/1/1	**10**
	Place cell bursts (per session)	(200,169,314)/(224,169,152,225)/123/-	**1576**	(105,63)/35/(104,55)/(123,168)/55/106/188	**1002**
POST	Sessions (per animal)	3/4/1/1	**9**	2/1/2/2/2/1/1	**11**
	Place cell bursts (per session)	(308,235,611)/(1741,1207,1062,423)/ 766/80	**6433**	(66,25)/50/(313/41)/(27/113)/(42/31)/ 46/562	**1316**

Blue numbers indicate animals that were also used for a prior publication (Schlesiger et al., 2015). Numbers from different animals are separated by "/". Burst numbers in parenthesis are from the same animal on different days. Bold numbers indicate the sum for each condition. Only sessions are included in this table where 20 or more place cell bursts occurred, which are the sessions that were used for sequence analysis

Supplementary Table 2. Number of sessions and co-activation patterns used in co-activation analysis.

	Control		Lesion	
Sessions (per animal)	3/3/3/1	**10**	3/3/4/4/4/1/0	**19**
Number of co-activation patterns (per session)	(7,3,6)/(5,6,6)/(4,6,3)/4	**50**	(4,5,4)/(5,5,4)/(4,1,4,1)/(5,4,4,5)/(4,3,4,2)/4/0	**72**

Conventions are the same as in Supplementary Table 1.

Supplementary Table 3. Coincidence between Co-activation peaks and place field bursts

	P(Burst|Co-act.)	p value	P(Replay|Co-act.)	p value
Control	0.34 +/- 0.07 (n = 18)	2.6 E-3	0.036 +/- 0.009 (n = 17)	2.4 E-3
MEC-Lesioned	0.13 +/- 0.05 (n = 24)		0.019 +/- 0.013 (n = 21)	

Coincidence (150 ms time window) between co-activation peaks and place field bursts (left) /significant replay (right) measured by estimated conditional probabilities P (PRE and POST epochs combined). Values are given as mean +/- sem. P-values for differences between Control and MEC-lesioned animals are derived from a ranksum test.

Supplementary Table 4. Regression Slope statistics for co-activation analysis

Control				MEC-Lesioned			
PRE-RUN		PRE-POST		PRE-RUN		PRE-POST	
Slope	p value	Slope	p value	Slope	p value	Slope	p value
1.7	<2e-05	1.2	<2e-05	1.46	<2e-05	1.075	0.007
1.45	<2e-05	1.23	<2e-05	1.09	0.0014	0.99	0.7
1.25	<2e-05	1.33	<2e-05	1.2	<2e-05	1.16	<2e-05
1.55	<2e-05	1.16	<2e-05	1.64	<2e-05	1.12	<2e-05
				1.09	0.0006	1.02	0.15
				1.13	<2e-05	1.09	0.003

Regression slopes from Figure S7a for all 4 control animals (rows) and all 6 MEC-lesioned animals. P values are obtained from the distribution of regression slopes obtained by 50,000 shuffles of cell indices (blue distributions in Fig. S7 a), where the regression line was fitted to as many data points as there were patterns in the real data of the specific animal. Red numbers indicate insignificant sessions.

Supplementary Figure 1.

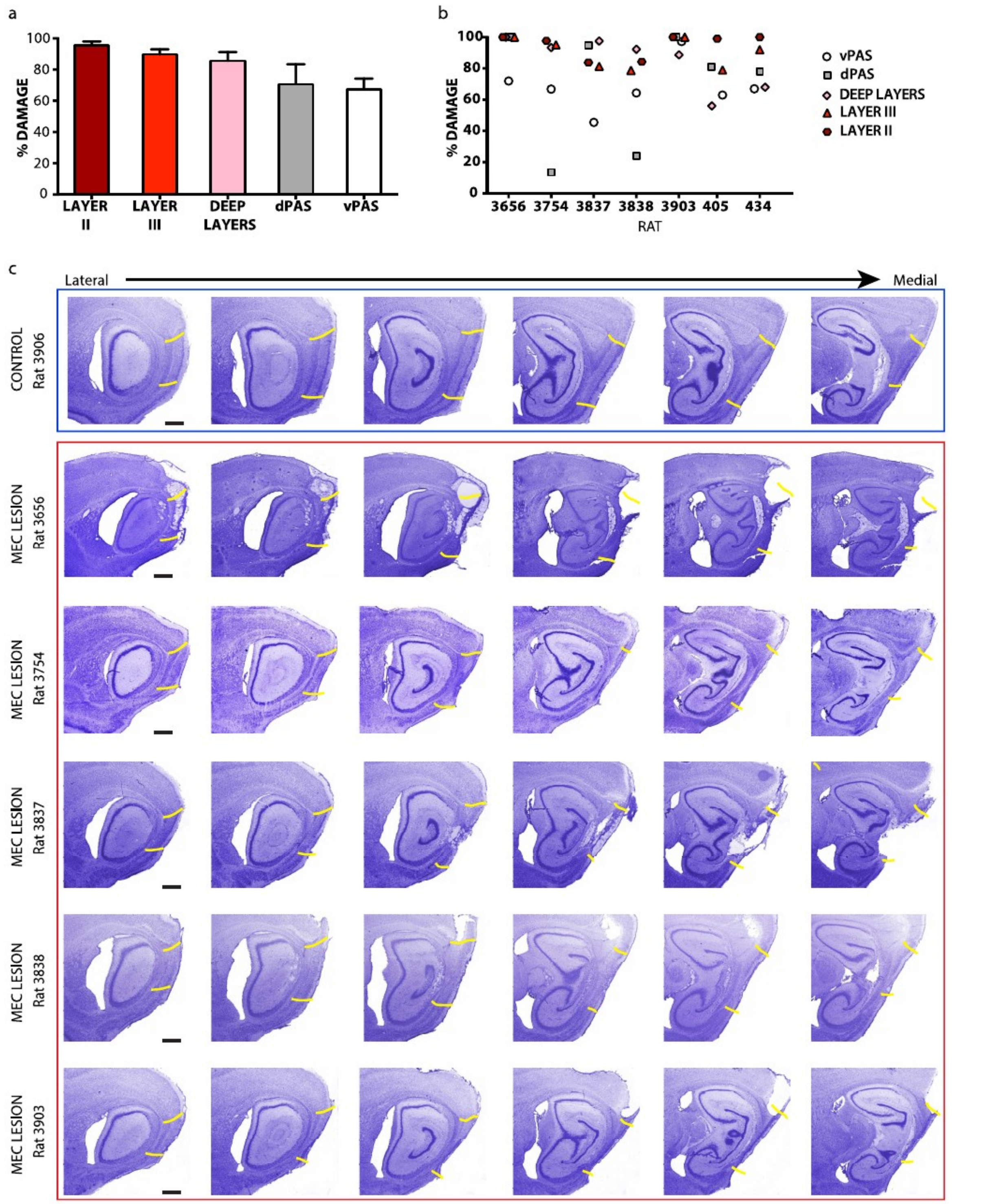

MEC lesions were nearly complete. (a) Average lesion sizes of layer II, layer III, deep layers (V/VI), dorsal parasubiculum (dPAS), and ventral parasubiculum (vPAS) were quantified separately (n = 7 animals). Error bars represent SEM. (b) Percentage of lesioned tissue for each of the rats included in the analysis. No significant correlations were found between lesion extent (layer II/III) and sequence replay measured by animal-wise mean fraction of significant replays (PRE: Pearson's r = -0.076, p = 0.90, n = 7 animals; RUN: r = 0.42, p = 0.35, n = 7 animals; POST: r = -0.13, p =

0.77, n = 7 animals). (c) Detailed illustration of series of sagittal sections. Scale bar, 1mm. First row correspond to a representative example of a control rat. The following 5 rows correspond to sagittal sections of 5 out of the 7 rats used for the analysis. Histology of the remaining two rats (405 and 434) can be found in Hales et al. 2014 [34].

Supplementary Figure 1 (Part 2)

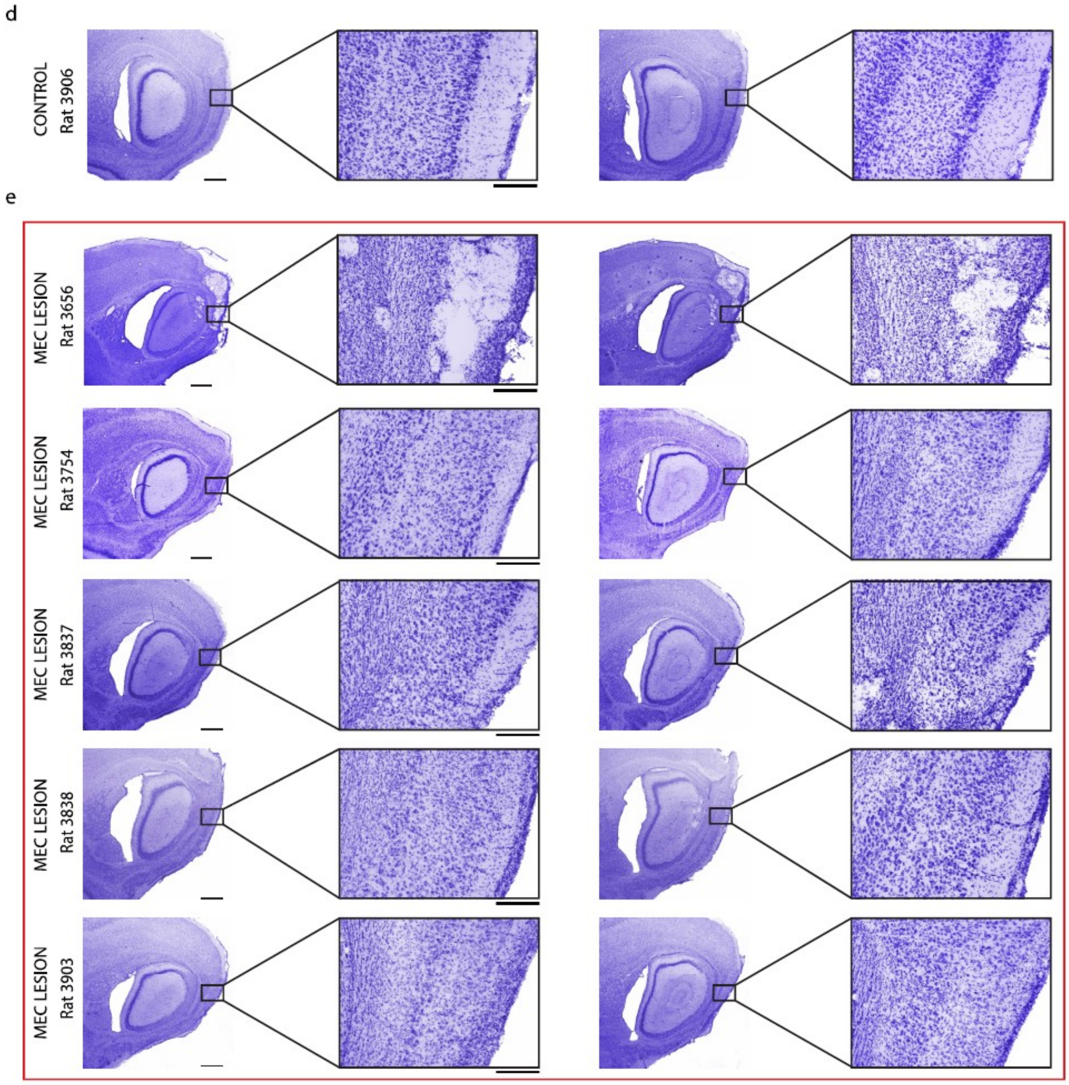

MEC lesions were nearly complete. (Continued) (d) Example of the two most lateral sagittal sections of a control rat with the delimited areas shown at a higher magnification. Scale bar, left panel, 1mm; right panel (inset), 250 µm. (e) Example of the two most lateral sections of 5 MEC lesion rats with the delimited areas of each section shown at a higher magnification. Scale bar, left panels, 1mm; right panels (inset), 250 µm. Even when cells were not completely necrotic in the most lateral part of the MEC, they lost their layer-specific organization and had a damaged appearance.

Supplementary Figure 2 (Part 1).

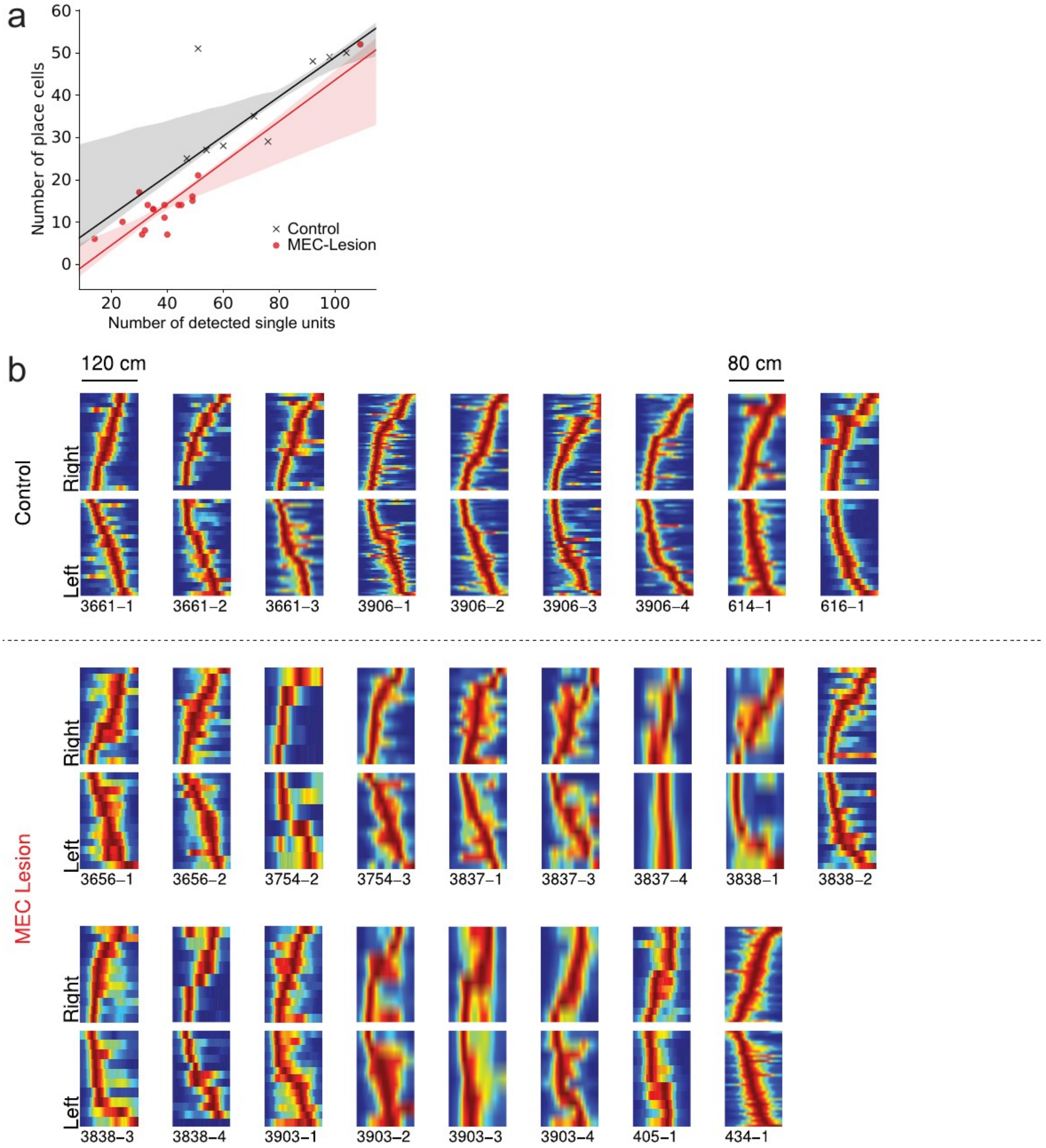

Place field templates and spatial similarity indices (SSIs). (a) Place cells vs. the total number of single units identified in the spike sorting process. In both the control and the MEC lesion group the number of place cells was about half the total number of recorded neurons, and the two numbers were highly correlated (Linear regression; Control: p = 2.0e-6, n = 9 sessions, Lesioned: p = 5.6e-8, n = 17 sessions; each symbol represents a recording session). (b) Place fields for all sessions (labels below) separated into rightward and leftward runs (each line shows a place cell's firing rate along the linear track). Fields are ordered according to the position of the peak firing rate. Sessions are indexed by “rat number-experimental session” (e.g. 614-1). The track length was 150 cm except for

rats 614, 616, 405, and 434 where it was 100 cm. Only 90% of the track length were used for analysis, excluding the reward locations.

Supplementary Figure 2 (Part 2)

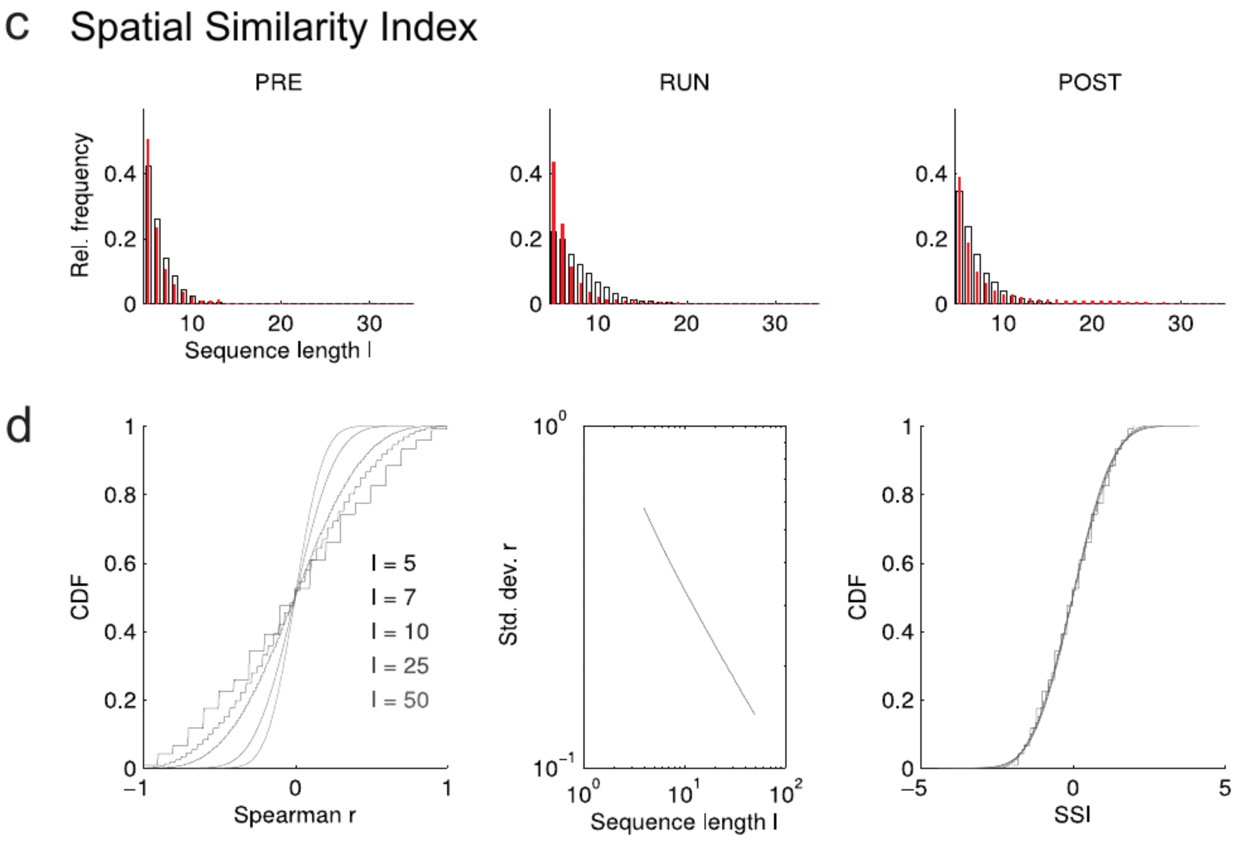

Place field templates and spatial similarity indices (SSIs). (Continued) (c) Distributions of sequence lengths from PRE, RUN, and POST conditions. Black: control animals. Red: MEC-lesioned animals. (d) Left: Cumulative distribution functions of rank order correlation coefficients for random sequences of length L (grey levels). Middle: Standard deviations from surrogate distributions on the left as a function of L. Right: CDFs for SSIs and different sequence lengths L (grey level). SSIs are rank order correlation coefficients scaled by the standard deviation from the middle graph. The cumulative distributions of SSIs are virtually sequence length independent.

Supplementary Figure 2 (Part 3)

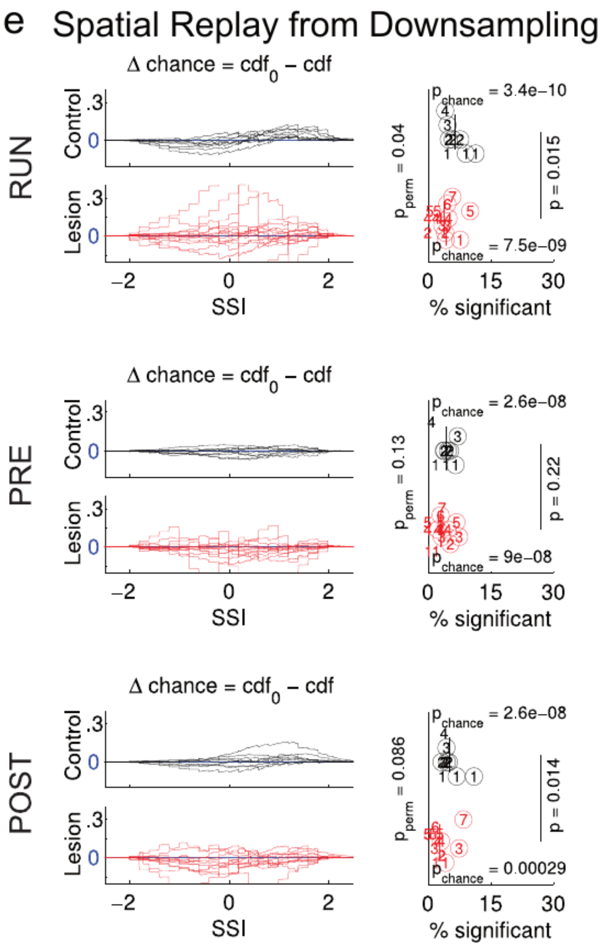

Place field templates and spatial similarity indices (SSIs). (Continued) (e) SSI statistics (as in Fig. 2c,d) for place cell bursts with 20 random downsamples each matching the number of place cells in the MEC-lesioned group (Ranksums for ranksum tests in the right column; RUN: $n_{Control} = 9$ epochs, $n_{Lesion} = 15$ epochs, r.s. = 154, PRE: $n_{Control} = 9$ epochs, $n_{Lesion} = 14$ epochs ,r.s. = 128, POST: $n_{Control} = 9$ epochs, $n_{Lesion} = 13$ epochs , r.s. = 141; Note that the numbers of observation are higher as indicated in Table S1, since 20 bootstrapping repetitions resulted in a higher numbers of bursts with 5 or more place cells, and we required more than 20 bursts to include an epoch into the analysis).

Supplementary Figure 3

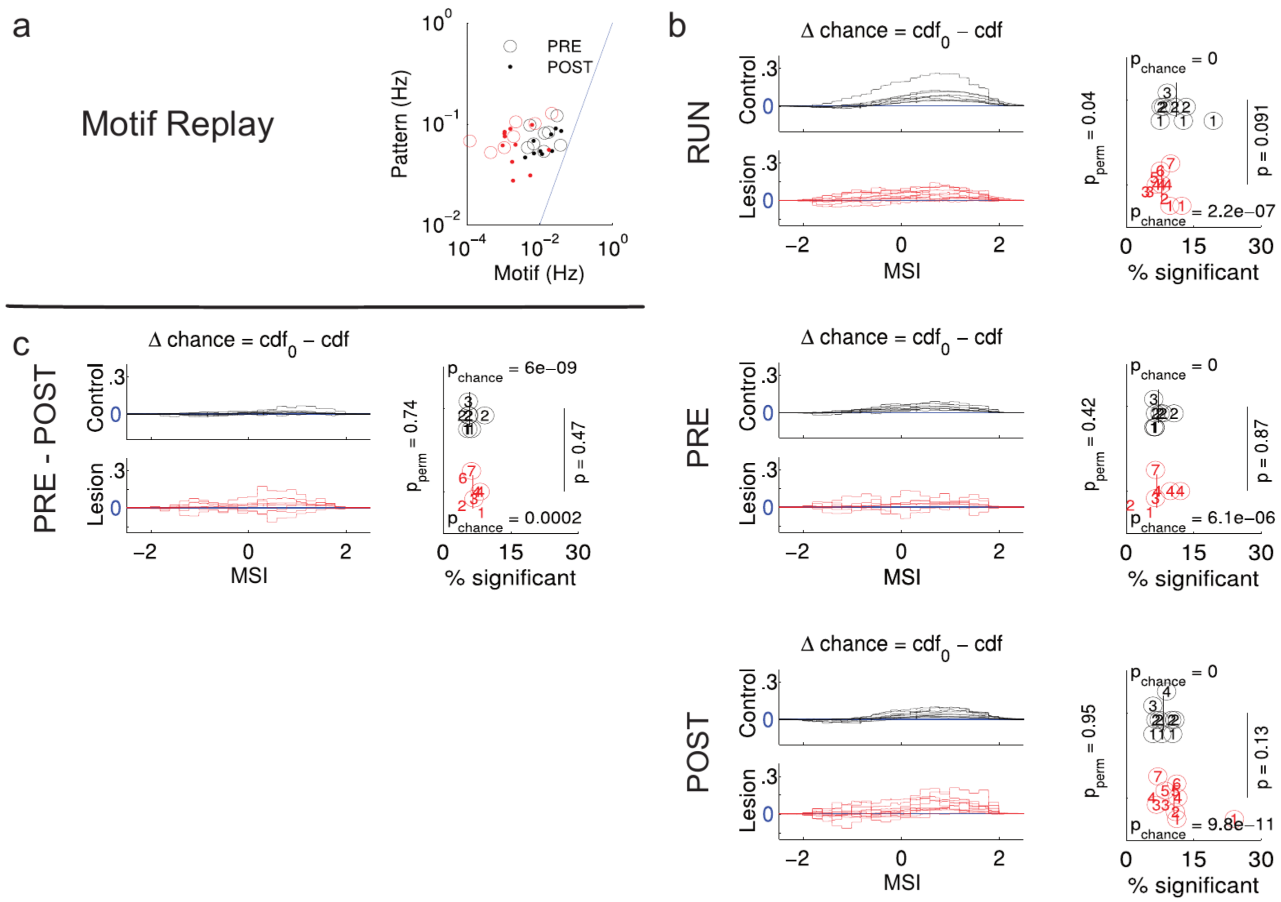

Motif similarity. (a) Same as in Fig. 2b and c for Motif bursts. Motif similarity indices (MSIs) measure the length normalized rank order correlation coefficients between all pairs of cell sequences in an epoch (Ranksums for ranksum tests in the right column; RUN: $n_{Control}$ = 8 epochs, n_{Lesion} = 11 epochs, r.s. = 101, PRE: $n_{Control}$ = 8 epochs, n_{Lesion} = 7 epochs, r.s. = 62, POST: $n_{Control}$ = 9 epochs, n_{Lesion} = 11 epochs, r.s. = 74; Note that the numbers of epochs are higher than indicated Table S1, since we had improved statistics by including rank order correlations not only between burst and two templates but between all pairs of bursts). (b) Similarity between motifs in PRE and POST session (ranksum = 57, $n_{Control}$ = 8 epochs, n_{Lesion} = 7).

Supplementary Figure 4

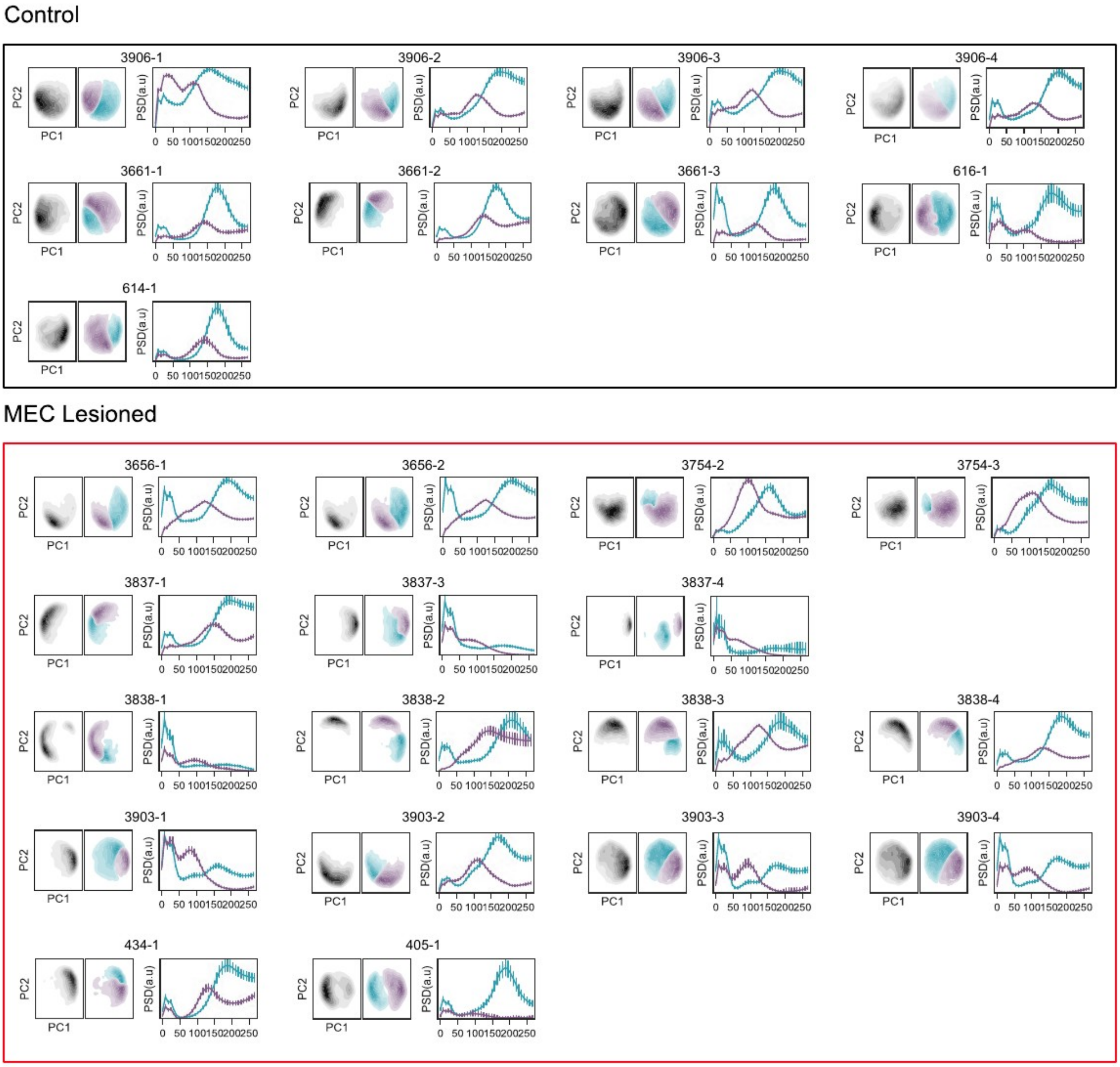

Field potential analysis. Clustering of high frequency events into SWRs and FGBs for all sessions (as labeled in Supplementary fig. 2b). Principal component (PC) coordinates and power spectra as in Fig. 5b and c. Note that the PCs were different between sessions and therefore the clustering appears different. However, the frequency distribution of the two types of events was consistent across sessions.

Supplementary Figure 5

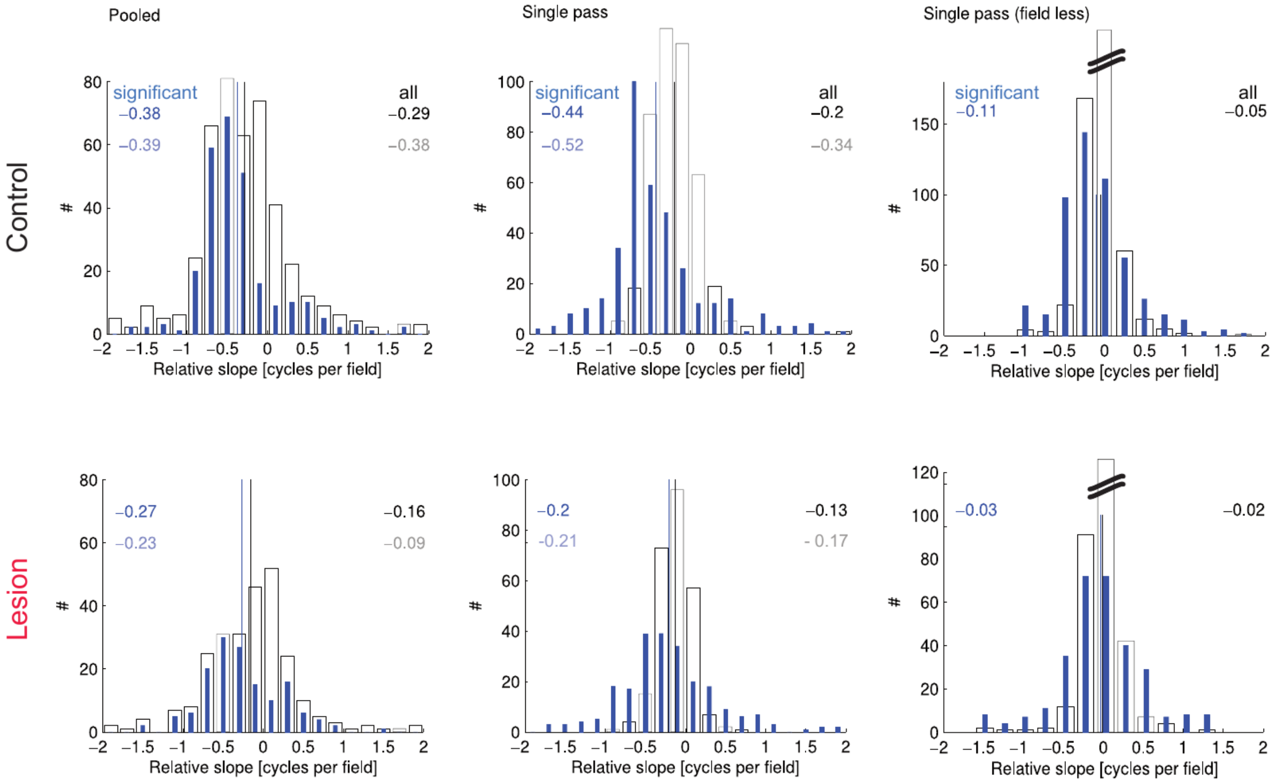

Phase precession analysis. Data from 2 of 4 animals (Rats 614, 616; Supplementary Figure 2) from the control group, and 2 out of 7 animals (Rats 405, 434; Supplementary Figure 2) from the MEC-lesioned group were already analyzed in our previous publication (Schlesiger et al. 2015). We repeated phase precession analysis for the data from the remaining 2 control (top) and 5 MEC-lesioned (bottom) animals to check for consistency with the previous data set. When pooled over all runs (left column) and in single runs (middle column; mean single run slope per field), precession slopes in unconstrained place field were comparable to those in the previous paper (numbers inside the plot indicate mean slopes for significant (dark blue) and all (black) phase-field correlations, lighter fonts indicate corresponding mean values from Schlesiger et al. 2015). For both pooled and single run slopes, phase precession was significantly higher in control animals than in MEC-lesioned animals (Pooled: p = 9.0e-5 $n_{Control}$ = 576 place cells, n_{Lesion} = 374 place cells ; Pooled significant: p = 0.0032, $n_{Control}$ = 576 place cells, n_{Lesion} = 374 place cells; Single run: p = 7.2E-6 $n_{Control}$ = 576 place cells, n_{Lesion} = 374 place cells, Single run significant p = 3.9 E-9, $n_{Control}$ = 576 place cells, n_{Lesion} = 374 place cells; ranksum tests). Phase precession analysis ways done as described in (Schlesiger et al.2015) only for place fields which had had spikes in at least 4 subsequent theta cycles. Phase precession analysis in unconstrained fields may pick up spurious negative slopes (Schlesiger et al. 2015) and indeed slopes were significantly negative for both animals groups (Control n = 576 place cells: Pooled p = 1.4e-27, Pooled significant p = 3.2e-24, Single run p =1.9e-40, Single run significant p = 2.6E-32; MEC-lesioned n = 364 place cells,

Pooled p = 2.2E-7, Pooled significant p = 9.4E-10, Single run p = 3.6E-19, Single run significant p = 5.7E-9; one-sided signed rank tests). We therefore also did phase precession analysis where we constrained the analysis to bursts of spikes (Right column). In such a field-less approach, only slopes are computed if spikes are not separated by three or more theta cycles without activity (right histogram, numbers inside the plot indicate mean slopes for significant (dark blue) and all (black) phase-field correlations). In control animals both mean significant slopes and mean slopes were significantly below 0 (p = 7.2 e-13 blue; p = 3.8 e-14 ; black, n = 576 place cells; one-sided signed rank tests), whereas in MEC-lesioned animals the slopes were not significantly different from 0 (p = 0.10 blue; p = 0.13 black; n = 374 place cells; one-sided signed rank tests). Again, slopes in the control group were significantly smaller than in the MEC-lesioned group (All slopes: p = 5.6e-5, $n_{Control}$ = 576 place cells, n_{Lesion} = 374 place cells; Significant slopes: p = 7.0 E-4, $n_{Control}$ = 576 place cells, n_{Lesion} = 374 place cells; ranksum tests). In summary, we observed substantially reduced phase precession in MEC-lesioned compared to control rats, as reported in the previous data set.

Supplementary Figure 6 (Part 1)

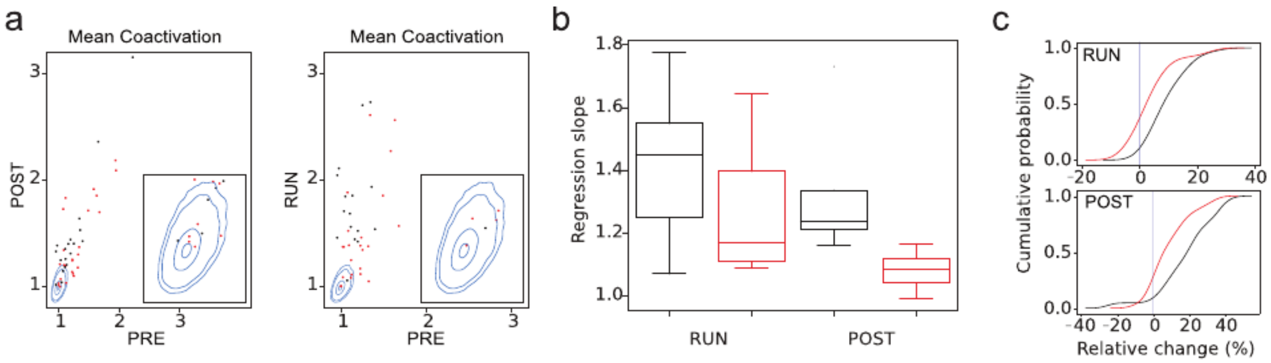

Animal-wise results. (a,b,c) Reproduction of Figure 1 panels e,f,g only using one session per animal (the one with maximal number of recorded units). (a) Despite lower number of coactivation patterns (Control: n=21; MEC-lesion: n=25), the mean co-activation is still above chance (as compared to surrogate data) level (Ranksum tests; Control: PRE p=6.5e-7, r.s. = 2305196; RUN p=2.0e-10, r.s. = 2552286; POST p=6.5e-14 r.s. = 2633220; Lesion: PRE p=1.5e-13, r.s. = 3126009; RUN p=4.0e-15, r.s. = 3217466; POST p=1.4e-14, r.s. = 3186151). Also correlations between PRE-POST and PRE-RUN remain significant (PRE-RUN control: Spearman's r = 0.51, p = 0.017, n = 21 patterns, PRE-POST Control: Spearman's r = 0.85, p = 1.4e-6 n = 21 patterns. MEC-Lesioned: Spearman's r = 0.67, p = 2.5e-4, n = 25 patterns for PRE-POST and Spearman's r = 0.64, p = 5.2e-4, n = 25 patterns for PRE-RUN). (b) All four groups still have regression slopes significantly larger than expected from surrogate data obtained by 50,000 shuffles of cell indices (Control: PRE-POST and PRE-RUN all 4 animals were significant, p = 0 binomial test; MEC-lesioned PRE-POST 4 out of 6 animals were significant, p = 1.8E-6; PRE-RUN: all 6 animals were significant, p = 0; see Table S4) (c) Relative changes in co-activation strengths remain significantly positive in all groups (signed rank tests, PRE-POST control: p = 0.0001, s.r. = 1.0, n = 20 patterns; MEC lesion p = 0.04, s.r. = 89.0, n = 25 patterns; PRE-RUN control: p = 8.0E-4, s.r. = 15, n = 20 patterns; MEC lesion p = 3.2E-3, s.r. = 53, n = 25 patterns). Also, these relative changes are still significantly different among the two experimental groups (rank sum tests, Control vs. Lesioned: PRE-RUN : p = 0.003, r.s. =129.0, n = 45 patterns, and PRE-POST change p = 0.004, r.s. = 133.0, n = 46 patterns).

Supplementary Figure 6 (Part 2)

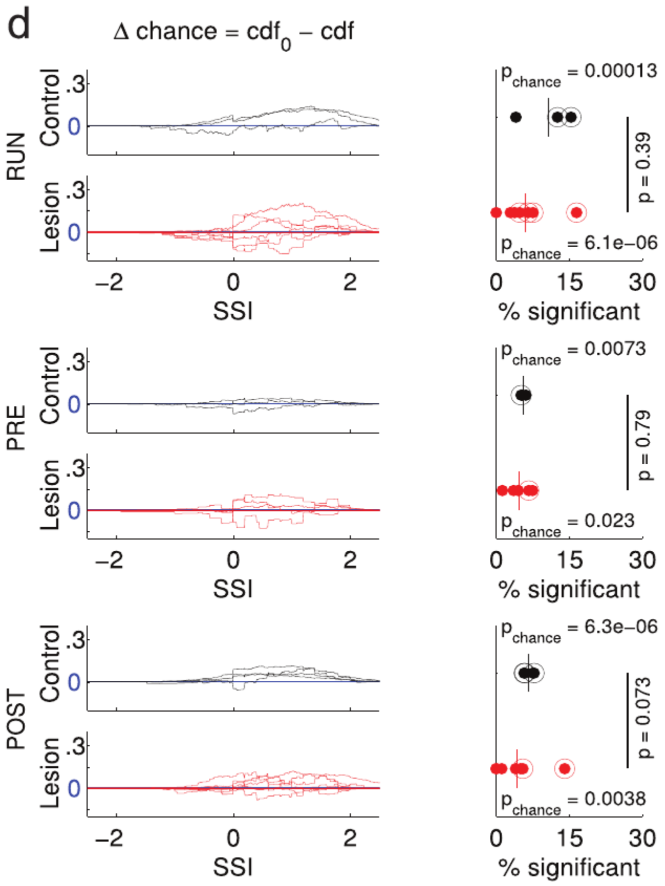

Animal-wise results. (Continued) (d) Reproduction of Figure 2 b,c combining all session per animal (n_{RUN} = 3 animals, n_{PRE}= 3 animals, n_{POST}=4 animals). RUN: 2/3 control animals and 4/7 MEC-lesioned animals showed significant replay (comparison of medians: ranksum = 21). PRE: 1/3 control animals and 1/5 MEC-lesioned animals showed significant replay (comparison of medians: ranksum = 15). POST: 3/4 control animals and 2/7 MEC-lesioned animals showed significant replay (comparison of medians: ranksum = 34).

Supplementary Figure 6 (Part 3)

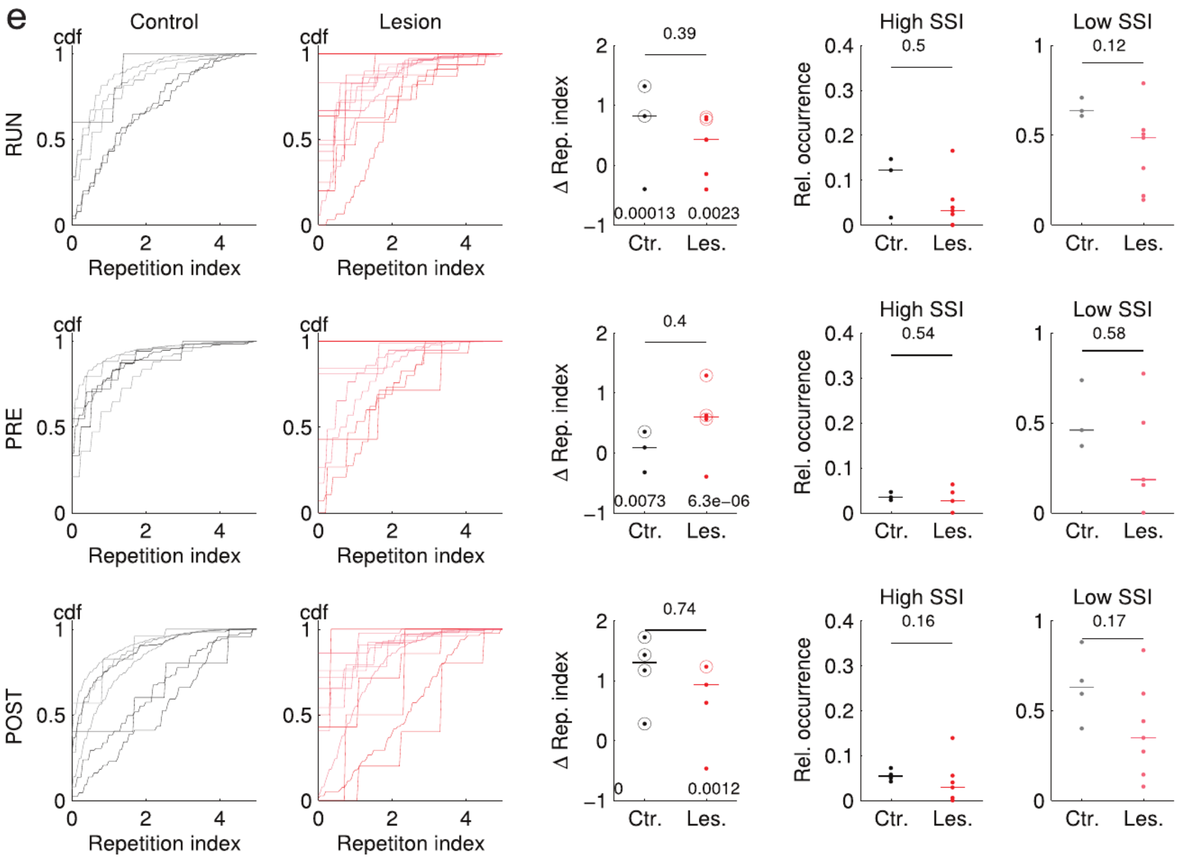

Animal-wise results. (Continued) (e) Reproduction of Figure 3 combining all session per animal. Stats for difference of medians (middle column; p-values above bar were obtained from ranksum test, RUN: r.s. = 19, PRE: r.s. = 9, POST: r.s.= 22; p values above group labels were obtained from a binomial test on the number of significant epochs (circles) for a chance level of 5%; RUN: 2 out of 3 animals were significant in control animals, 2 out of 6 animals were significant in MEC-lesioned animals; PRE: 1 out of 3 animals were significant in control animals, 3 out of 4 animals were significant in MEC-lesioned animals; POST: 4 out of 4 animals were significant in control animals, 2 out of 5 animals were significant in MEC-lesioned animals; colors as in a) and b). In the two rightmost columns, relative occurrences are compared by ranksum tests, RUN high: r.s. = 20, RUN low: r.s. = 24; PRE high: r.s. = 16, PRE low: r.s. = 16; POST high: r.s.= 32, POST low: r.s. = 32.

Supplementary Figure 6 (Part 4)

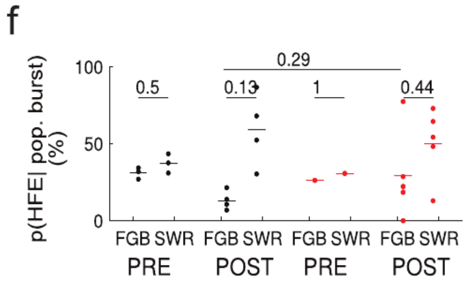

Animal-wise results. (Continued) (f) Reproduction of Figure 5 f combining all session per animal. P values above bars were obtained from signed rank tests (Control PRE: s.r. = 1, n = 3 animals; Control POST: s.r.= 0, n = 4 animals; MEC-lesioned PRE: s.r. = 0, n = 1 animal; MEC-lesioned POST: s.r. = 4, n = 5 animals). Ranksum test between control and MEC-lesioned animals (upper black bar) for FGB POST has ranksum 15.
