# Supplementary material for: Hippocampal CA1 replay becomes less prominent but more rigid without inputs from medial entorhinal cortex
Source: Nat Commun. 2019 Mar 22;10:1341. doi: 10.1038/s41467-019-09280-0 (PMC6430812; doi:10.1038/s41467-019-09280-0)
Supplement: Supplementary file 2 — Reporting Summary [file 41467_2019_9280_MOESM2_ESM.pdf]

nature research

Corresponding author(s): Christian Leibold

Last updated by author(s): Feb 22, 2019

Reporting Summary

Nature Research wishes to improve the reproducibility of the work that we publish. This form provides structure for consistency and transparency in reporting. For further information on Nature Research policies, see Authors & Referees and the Editorial Policy Checklist.

Statistics

For all statistical analyses, confirm that the following items are present in the figure legend, table legend, main text, or Methods section.

n/a	Confirmed	
☐	☒	The exact sample size (n) for each experimental group/condition, given as a discrete number and unit of measurement
☐	☒	A statement on whether measurements were taken from distinct samples or whether the same sample was measured repeatedly
☐	☒	The statistical test(s) used AND whether they are one- or two-sided<br>*Only common tests should be described solely by name; describe more complex techniques in the Methods section.*
☐	☒	A description of all covariates tested
☐	☒	A description of any assumptions or corrections, such as tests of normality and adjustment for multiple comparisons
☐	☒	A full description of the statistical parameters including central tendency (e.g. means) or other basic estimates (e.g. regression coefficient) AND variation (e.g. standard deviation) or associated estimates of uncertainty (e.g. confidence intervals)
☐	☒	For null hypothesis testing, the test statistic (e.g. F, t, r) with confidence intervals, effect sizes, degrees of freedom and P value noted<br>*Give P values as exact values whenever suitable.*
☒	☐	For Bayesian analysis, information on the choice of priors and Markov chain Monte Carlo settings
☐	☒	For hierarchical and complex designs, identification of the appropriate level for tests and full reporting of outcomes
☐	☒	Estimates of effect sizes (e.g. Cohen's d, Pearson's r), indicating how they were calculated

Our web collection on statistics for biologists contains articles on many of the points above.

Software and code

Policy information about availability of computer code

Data collection	Recordings were performed using Cheeta 5.0 software (Neuralynx , https://neuralynx/software/cheetah-5.0-legazy).
Data analysis	Spike sorting was performed using Klustakwik 2.0.1-1 (https://klusta.readthedocs.io/en/latest/) and Mclust 3.5 (http://redishlab.neuroscience.umn.edu/Mclust/Mclust.html) as described in the Methods section.<br>Further analysis was performed with custom MATLAB (R2013b,R2015b,R2018b, Mathworks) and Python 2.7 code. The custom MATLAB core routines for sequence analysis are available from the github repository: https://github.com/cleibold/ReactivationCode. All other analysis code is available on request.

For manuscripts utilizing custom algorithms or software that are central to the research but not yet described in published literature, software must be made available to editors/reviewers. We strongly encourage code deposition in a community repository (e.g. GitHub). See the Nature Research guidelines for submitting code & software for further information.

Data

Policy information about availability of data

All manuscripts must include a data availability statement. This statement should provide the following information, where applicable:

- Accession codes, unique identifiers, or web links for publicly available datasets
- A list of figures that have associated raw data
- A description of any restrictions on data availability

The recording data are available upon request to the corresponding author.

Field-specific reporting

Please select the one below that is the best fit for your research. If you are not sure, read the appropriate sections before making your selection.

☒ Life sciences ☐ Behavioural & social sciences ☐ Ecological, evolutionary & environmental sciences

For a reference copy of the document with all sections, see nature.com/documents/nr-reporting-summary-flat.pdf

Life sciences study design

All studies must disclose on these points even when the disclosure is negative.

Sample size	No statistical methods were used to predetermine sample sizes, but our sample sizes are comparable to those reported in previous publications.
Data exclusions	An exclusion criteria was pre-established at the time of the experimental design. Animals from the MEC lesion group with sparing tissue of more than 30% of layer II or layer III were excluded from the experiment
Replication	All acquired data derived from the described experiments were included in this study. The data sample is thus unique and replication was not possible.
Randomization	Animals were randomly assigned to lesion and control group before surgery.
Blinding	Researchers were not blind to group assignment

Reporting for specific materials, systems and methods

We require information from authors about some types of materials, experimental systems and methods used in many studies. Here, indicate whether each material, system or method listed is relevant to your study. If you are not sure if a list item applies to your research, read the appropriate section before selecting a response.

Materials & experimental systems

n/a	Involved in the study	
☒	☐	Antibodies
☒	☐	Eukaryotic cell lines
☒	☐	Palaeontology
☐	☒	Animals and other organisms
☒	☐	Human research participants
☒	☐	Clinical data

Methods

n/a	Involved in the study	
☒	☐	ChIP-seq
☒	☐	Flow cytometry
☒	☐	MRI-based neuroimaging

Animals and other organisms

Policy information about studies involving animals; ARRIVE guidelines recommended for reporting animal research

Laboratory animals	Long-evans male rats were used between 4-8 months of age.
Wild animals	*Provide details on animals observed in or captured in the field; report species, sex and age where possible. Describe how animals were caught and transported and what happened to captive animals after the study (if killed, explain why and describe method; if released, say where and when) OR state that the study did not involve wild animals.*
Field-collected samples	*For laboratory work with field-collected samples, describe all relevant parameters such as housing, maintenance, temperature, photoperiod and end-of-experiment protocol OR state that the study did not involve samples collected from the field.*
Ethics oversight	All animal experiments were approved by the University of California, San Diego Institutional Animal Care and Use Committee and conducted according to National Institute of Health Guidelines

Note that full information on the approval of the study protocol must also be provided in the manuscript.
